# Subtyping of microsatellite stability colorectal cancer reveals guanylate binding protein 2 (GBP2) as a potential immunotherapeutic target

**DOI:** 10.1136/jitc-2021-004302

**Published:** 2022-04-05

**Authors:** Haizhou Wang, Yabo Zhou, Yangyang Zhang, Shilin Fang, Meng Zhang, Haiou Li, Fei Xu, Lan Liu, Jing Liu, Qiu Zhao, Fan Wang

**Affiliations:** 1 Department of Gastroenterology, Zhongnan Hospital of Wuhan University, Wuhan, China; 2 Hubei Clinical Center and Key Lab of Intestinal and Colorectal Diseases, Wuhan, China; 3 Department of Immunology & National Key Laboratory of Medical Molecular Biology, Institute of Basic Medical Sciences, Chinese Academy of Medical Sciences (CAMS) & Peking Union Medical College, Beijing, China; 4 Department of Pain, Renmin Hospital of Wuhan University, Wuhan, China

**Keywords:** Immunotherapy, Biomarkers, Tumor, Gene Expression Profiling, Immunologic Surveillance

## Abstract

**Backgrounds:**

Proficient-mismatch-repair or microsatellite stability (pMMR/MSS) colorectal cancer (CRC) has limited efficacy for immune checkpoint blockade (ICB) therapy and its underlying mechanism remains unclear. Guanylate binding protein 2 (GBP2) is a member of the GTPase family and is crucial to host immunity against pathogens. However, the correlations between GBP2 and immunosurveillance and immunotherapy for pMMR/MSS CRC have not been reported.

**Methods:**

Unsupervised clustering was employed to classify immune class and non-immune class in 1424 pMMR/MSS patients from six independent public datasets. This binary classification was validated using immune cells or response related signatures. The correlation between GBP2 and immune microenvironment was explored using well-established biological algorithms, multiplex immunohistochemistry (mIHC), in vitro and in vivo experiments.

**Results:**

We classified 1424 pMMR/MSS CRC patients into two classes, ‘immune’ and ‘non-immune’, and GBP2 was identified as a gene of interest. We found that lower GBP2 expression was correlated with poor prognosis and metastasis. GBP2 expression was also upregulated in the immune class and highly associated with interferon-γ (IFN-γ) signaling pathway and CD8 +T cell infiltration using gene set enrichment analysis, gene ontology analysis, single-cell sequencing and mIHC. Moreover, reduced GBP2 expression inhibited the antigen processing and presentation machinery and CXCL10/11 expression in MSS CRC cells on IFN-γ stimulation. A Transwell assay revealed that deletion of GBP2 in murine MSS CRC cells reduced CD8 +T cell migration. Mechanistically, GBP2 promoted signal transducer and transcription activator 1 (STAT1) phosphorylation by competing with SHP1 for binding to STAT1 in MSS CRC cells. Finally, an unsupervised subclass mapping (SubMap) algorithm showed that pMMR/MSS patients with high GBP2 expression may correlate with a favorable response to anti-PD-1 therapy. We further confirmed that GBP2 knockout reduced CD8 +T cell infiltration and blunted the efficacy of PD-1 blockade in tumor-bearing mice.

**Conclusions:**

Our study reveals that pMMR/MSS CRC is immunogenically heterogeneous and that GBP2 is a promising target for combinatorial therapy with ICB.

**Key messages**

**What is already known on this topic**

- Monotherapy of immune checkpoint blockade (ICB) is ineffective for proficient-mismatch-repair or microsatellite stability (pMMR/MSS) colorectal cancer (CRC) patients. Combination of ICB with other therapies to improve antitumor activity of pMMR/MSS CRC is ongoing. There is an urgent need to investigate prognostic and predictive biomarkers to improve ICB response in pMMR/MSS CRC.

**What this study adds**

- In pMMR/MSS CRC, loss of guanylate binding protein 2 (GBP2) can promote immune escape by inhibiting antigen processing and presentation machinery and CXCL10/11 expression and reduce CD8 +T cell infiltration, thereby blunting the efficacy of ICB.

**How this study might affect research, practice or policy**

- Our findings prove for the first time that GBP2 potentially serves as a therapeutic target for sensitizing the ICB therapy in pMMR/MSS CRC.

## Introduction

Colorectal cancer (CRC) ranks third in terms of mortality worldwide.^1^ Approximately 15% of CRCs are deficient-mismatch-repair or microsatellite instability (dMMR/MSI), and the remaining 85% are proficient-MMR or microsatellite stability (pMMR/MSS).^2^ Meanwhile, the development of immune checkpoint blockade (ICB) has revolutionized the field of anticancer therapeutic intervention in recent years, involving the use of the checkpoint inhibitors targeting programmed cell death 1 (PD-1), programmed cell death-ligand 1 (PD-L1), or cytotoxic T lymphocyte antigen 4 (CTLA4).^3^ However, not all CRC patients benefit from ICB.

PD-1 antibodies (pembrolizumab and nivolumab) were approved by the Food and Drug Administration (FDA) for second-line treatment of dMMR/MSI-H CRC in 2017.^4^ Nevertheless, the prevailing view was that the monotherapy (eg, pembrolizumab) was ineffective for pMMR/MSS CRC patients before 2020.^5^ In these tumors, lack of immune cell infiltration and low tumor mutation burden (TMB) have been suggested as mechanisms of immune resistance.^4^ Therefore, to overcome these problems, several new combinatorial regimens in pMMR/MSS CRC are ongoing. After 2020, Chalabi *et al* reported that 4 of 27% (4/15) pMMR CRC patients showed pathological responses, with three major pathological responses (≤10% residual viable tumor) and one partial response under combination therapy with ipilimumab and nivolumab.^6^ A randomized phase two study also found that compared with best supportive care alone, MSS CRC patients treated with durvalumab and tremelimumab had significantly improved overall survival (OS), especially those with higher TMB.^7^ In addition, a recent phase Ib clinical trial study found that when MSS CRC patients were treated with nivolumab combined with regorafenib, the objective response rate reached 33%.^8^ These studies suggest that combination with other drugs with different mechanisms of action can potentially overcome resistance. Therefore, a better understanding of the resistance mechanism to improve the efficacy of ICB in pMMR/MSS CRC are urgently needed.

To better clarify resistance mechanism, the classification of tumors into ‘cold’ and ‘hot’ categories has increasingly advocated.^9^ The characteristics of hot tumors are high numbers of tumor-infiltrating lympanhocytes (TILs), high expression of immune checkpoints, possible genomic instability and the existence of pre-existing antitumor immune responses. In contrast, cold tumors are characterized by poor TIL infiltration, immunological ignorance (sparse immune checkpoint expression), high proliferation capacity, low TMB, and low expression of antigen processing and presentation machinery (APM).^9^ This binary classification has become a powerful concept for predicting the ICB response. Accordingly, MSI CRC shows higher levels of TILs and TMB and higher expression of immune checkpoints.^10^ In contrast, MSS CRC is considered as a less inflamed immune microenvironment.^10^ Therefore, dMMR/MSI CRC has achieved more compelling clinical data for ICB treatment compared with pMMR/MSS CRC. However, the underlying molecular mechanism distinguishing ‘cold’ and ‘hot’ MSS CRC remains unclear.

In this article, we classified 1424 pMMR/MSS CRC patients into two classes, ‘immune cold’ and ‘immune hot’, based on the unsupervised clustering, and guanylate binding protein 2 (GBP2) was identified as a gene of interest. GBP2 is a member of the GTPase family and is crucial to host immunity against pathogens.^11^ GBP2 is also used as a marker of interferon (IFN) responsiveness, because it is one of the most highly expressed genes after IFN-γ stimulation. In CRC, only one study has demonstrated that upregulating of GBP2 could inhibit the growth of CRC and increase the sensitivity of paclitaxel-resistant CRC cells to paclitaxel.^12^ The current study sheds light on the high correlation of GBP2 with high CD8 +T cell infiltration, APM and chemokine (C-X-C motif) ligand 10/11 (CXCL10/11) expression, and better PD-1 blockade response through several well-established biological algorithms, in vitro and in vivo experiments. Therefore, our study reveals that GBP2 could serve as a new immune therapeutic target for combinatorial therapy with ICB.

## Methods and materials

### pMMR/MSS dataset collection

In this study, six independent cohorts were selected, including a total of 1424 pMMR/MSS and 457 dMMR/MSI CRC patients: GSE39582; colon adenocarcinoma (COAD) from The Cancer Genome Atlas; GSE41258; GSE26682; pooled cohort one and pooled cohort 2. The upper quartile fragments per kilobase of transcript per million mapped reads (FPKM-UQ) for COAD were downloaded from the UCSC Xena browser (https://xenabrowser.net/datapages/). The remaining five cohorts were all downloaded from the Gene Expression Omnibus database (http://wwwncbinlmnih.gov/geo/). Pooled cohort 1 consisted of GSE4554, GSE13067, GSE13294, GSE18088 and GSE75316. Pooled cohort 2 consisted of GSE35896 and GSE39084. The ‘sva’ R package was used to remove batch effects for the pooled cohorts. Principal components analysis (PCA) was used to detect the results by ‘prcomp’ R function (online supplemental figure S1). Detailed information and the clinical phenotypes of all the six cohorts are shown in online supplemental tables S1 and S2. The common molecular subtypes (CMS) subtype annotation of COAD and GS39582 was provided by the Colorectal Cancer Subtyping Consortium.

10.1136/jitc-2021-004302.supp1 Supplementary data

10.1136/jitc-2021-004302.supp2 Supplementary data

10.1136/jitc-2021-004302.supp3 Supplementary data

### Classification and characterization of molecular subtypes

The ‘ConsensusClusterPlus’ R package was used for subtype identification based on the expression matrix.^13^ Before performing unsupervised clustering, we selected the genes with a high median absolute deviation (MAD) value (MAD >0.5) in the GSE39582 cohort. A total of 6238 genes were included for clustering with 80% item resampling, a maximum K (cluster number) of 4 and 1000 iterations. The optimal K and their stability were evaluated using the consensus clustering algorithm.^14^ PCA plot was used to detect differences in expression between the groups. The other five cohorts were also used to recapitulate the two subtypes after applying similar gene ordering. Previously reported immune-related gene signatures calculated using the single-sample gene-set enrichment analysis (ssGSEA) algorithm were used to characterize immune and non-immune subtypes (online supplemental table S3).^15^ Detailed immune-related bioinformatic analysis used in this study were described in online supplemental information.

10.1136/jitc-2021-004302.supp4 Supplementary data

### Cell culture and reagents

Two human MSS CRC cell lines (HT29 and SW480) and a murine MSS CRC cell line (CT26) were obtained from the China Center for Type Culture Collection (Wuhan, China). HT29 and SW480 cells were cultured in RPMI 1640 (Hyclone, USA) and CT26 cells were cultured in DMEM (Hyclone, USA) at 37°C with 5% CO2. The medium was supplemented with 10% fetal bovine serum (Gibco, Australia) and 1% penicillin-streptomycin. Human recombinant IFN-γ was purchased from PeproTech (300-02). An in vivo murine anti-PD-1 antibody (BE0146, USA) was purchased from BioXCell. Sodium orthovanadate (Na_3_VO_4_) were purchased from MACKLIN (S817660-25g).

### Animal model

The 6–8 weeks old female BALB/c mice and NOD.Cg-*Prkdc*^scid^ *Il2rg*^tm1Wjl^/SzJ (NSG) mice were purchased from the GemPharmatech and housed under specific pathogen free conditions in the Animal Experiment Center, Zhongnan Hospital of Wuhan University. Vec and GBP2 KO CT26 cells (1×10^6^ /mL) were subcutaneously injected into BALB/c and NSG mice. When the tumors had grown to 125 mm^3^, the BALB/c mice were randomized into Vec+IgG, Vec+anti-PD1, GBP2 KO +IgG, and GBP2 KO +anti-PD-1 groups. The anti-PD-1 antibody (200 µg per mouse) and IgG isotype were administered intratumorally every 3 days until the end of the observation period (day 30). Tumor volume (mm^3^) was measured and calculated as follows: formula: V=a×b×b/2. All procedures involving mice and research protocols were approved by the Experimental Animal Welfare Ethics Committee, Zhongnan Hospital of Wuhan University (No. ZN2021038).

### Transwell migration assay

CD8a microbeads (Miltenyi Biotec) were used to separate CD8 lymphocytes from 6 to 8 weeks old Balb/c mice. They were then stimulated with anti-CD3/CD28 Dynabeads (Thermo Fisher Scientific) at a 1:1 beads/cells ratio and 100 U/mL human IL-2 (PeproTech) for 96 hours. Transwell inserts with a pore size of 5.0 m. 5×10^5^ CD8 T cells were put into the top chamber of the inserts (5.0 µm pore size, Corning). The bottom of the bottom well was filled with IMDM medium or conditioned medium (CM) from CT26 control or GBP2-KO cells, with or without CXCL10/11 overexpression. In order to block CXCR3, CD8 T cells were first incubated with 10 µg/mL of anti-CXCR3 (BioLegend, 126517) for 30 min before being added to the top chamber. Plates were kept at 37°C overnight, and the contents of the lower chamber were taken. Trypan blue was used to count the number of CD8 +T cells that were still alive. Detailed information of other experiments protocols was also described in online supplemental information.

### Statistical analysis

Analysis was conducted using the GraphPad V.7.0 software and R V.3.6.1 software. Unpaired two-tailed Student’s t-test or one-way analysis of variance test were used for comparison between two or multiple groups, respectively. When the data was non-normally distributed, the Mann-Whitney test or Dunn’s test were used. Kaplan-Meier survival curves were plotted using the ‘‘survival’’ package and analyzed by the log-rank test. The correlation between GBP2 and clinicopathologic features was analyzed using the χ^2^ test. The results are expressed as the mean±SEM at least three replicates. All results were considered to be statistically significant at p<0.05.

## Results

### An immune class exists in pMMR/MSS CRC

**Figure 1:**
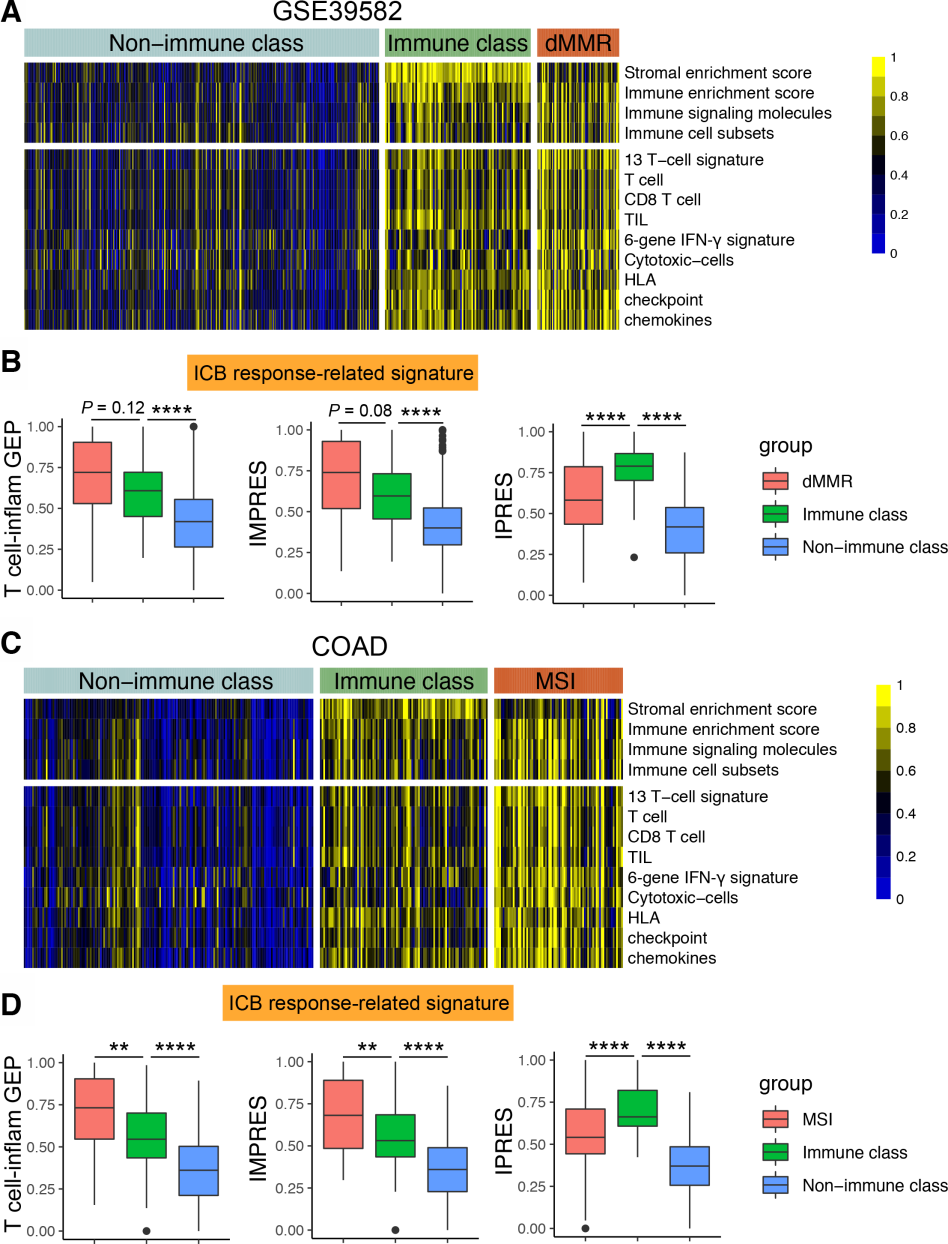
Identification of two distinct tumor microenvironment-based immune classes in pMMR/MSS CRC. An immune class and a non-immune class were identified using consensus clustering in the (A) GSE39582 cohort and (C) COAD cohort. In the heatmap, high and low ssGSEA scores of 13 immune cell or immune response signatures are represented in yellow and blue, respectively. Box plots showing expression of ICB response-related signatures between immune class, non-immune class and dMMR/MSI samples in the (B) GSE39582 cohort and (D) COAD cohort. **P<0.01, ****p<0.0001 vs control group. COAD, colon adenocarcinoma; CRC, colorectal cancer; dMMR/MSI, deficient-mismatch-repair or microsatellite instability; GEP, gene expression profile; ICB, immune checkpoint blockade; pMMR/MSS, proficient-mismatch-repair/microsatellite stability; ssGSEA, single-sample gene-set enrichment analysis.

To explain why a minority of MSS CRC patients respond to anti-PD-1 therapy,^8 16^ we stratified the MSS CRC patients by applying the consensus clustering approach based on gene expression in the six independent pMMR/MSS CRC cohorts. Two distinct classes (cluster-1 and cluster-2) were identified based on the 6238 RNAs with median absolute deviation (MAD) >0.5 (online supplemental figure S2 and S3a, c, e, g and table S7). We found that the patients in one of the clusters exhibited significant enrichment of signatures identifying immune cells or immune response. Meanwhile, a high similarity between MSI tumor and highly infiltrated MSS tumor were also observed (figure 1A, C and online supplemental figure S3a, c, e, g). Therefore, we termed the two classes as the immune class (IC) and non-immune class (non-IC). PCA analysis also found a robust difference of the expression profiles between IC and non-IC (online supplemental figure S2). Furthermore, compared with non-IC, higher enrichment scores of T cell inflamed gene expression profile (GEP), innate anti-PD-1 resistance (IPRES) and immuno-predictive score (IMPRES) signatures were observed in IC and MSI groups (figure 1B, D and online supplemental figure S3b, d, f). These signatures could predict the tumor response to ICB in melanoma.^17–19^ Taken together, these data indicate that immunological heterogeneity exists in the pMMR/MSS CRC samples.

10.1136/jitc-2021-004302.supp5 Supplementary data

### GBP2 expression is upregulated in IC

**Figure 2:**
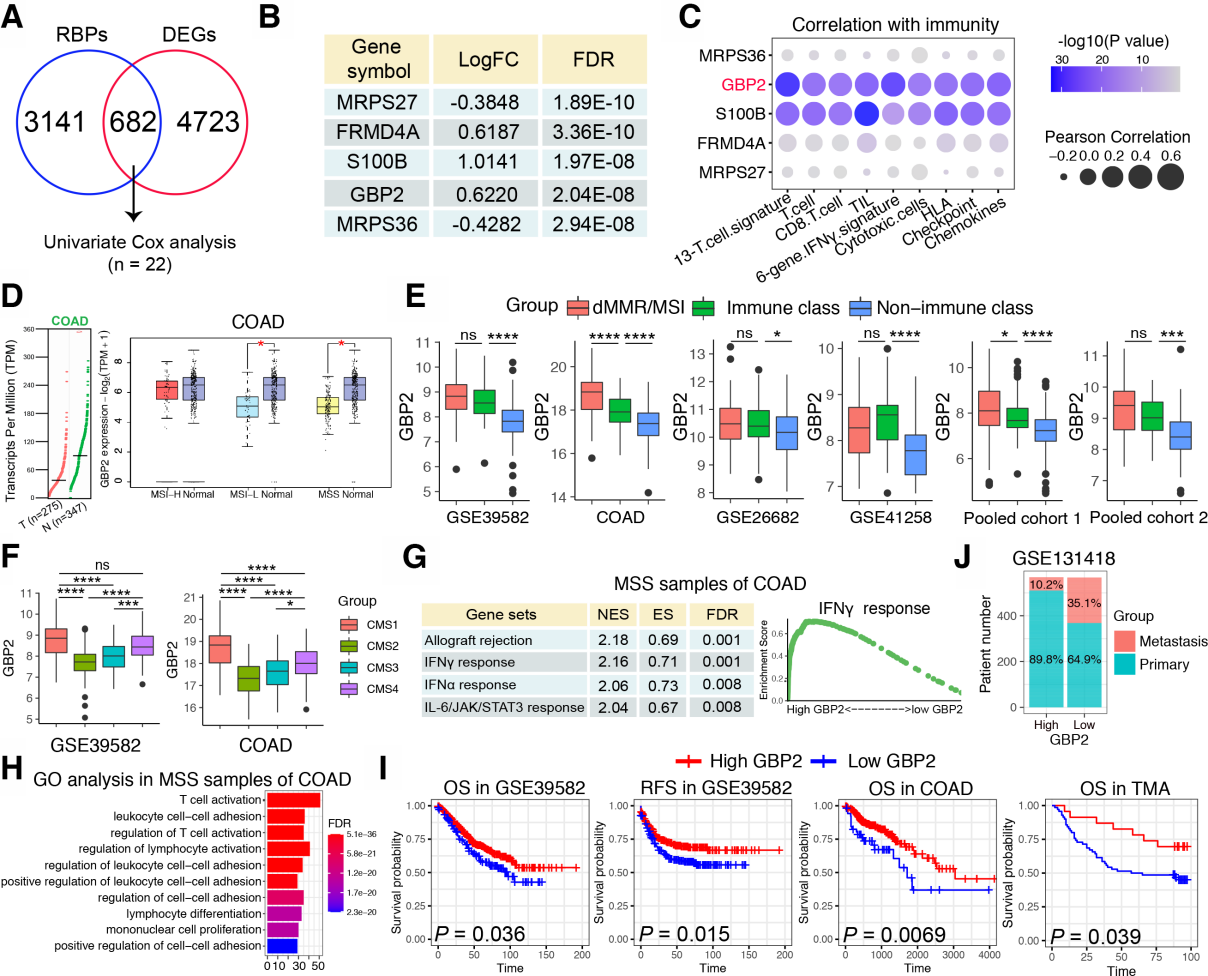
Identification of GBP2 as a gene of interest. (A) Venn diagram of intersection of differentially expressed genes (DEGs) and RNA binding proteins (RBPs) in the MSS samples of COAD cohort. The intersected genes were further screened by Univariate Cox analysis. The cut-off criteria of DEGs were log2 fold change >0.585 and an adjusted p<0.05. (B) Table showing top five genes that were significantly upregulated in the immune class in the MSS samples of COAD cohort. (C) Bubble plot representation showing the correlation between top 5 genes and 13 immune cell or immune response signatures in the MSS samples of COAD cohort. (D) The left panel shows GBP2 expression between 275 CRC specimens and 347 normal adjacent tissues from the The Cancer Genome Atlas (TCGA). The right panel showed GBP2 expression between MSI-H, MSI-L and MSS and normal tissues, respectively. The data were derived from the public Gene Expression Profiling Interactive Analysis2 (GEPIA2) data portal (http://gepia2.cancer-pku.cn). (E) Box plots showing expression of GBP2 between immune class, non-immune class and dMMR/MSI samples in the six cohorts. (F) Box plots showing expression of GBP2 between four CMS subtypes in the GSE39582 cohort and COAD cohort. (G) Table showing significant functional gene sets enriched in the MSS samples of COAD cohort using gene set enrichment analysis (GSEA). (H) GO analysis based on the top 200 differential expressed genes that were screened between high vs low GBP2 expression separated by median expression of GBP2 in the MSS samples of COAD cohort. (I) Survival analysis showing the relationship between GBP2 levels and the OS and RFS of samples in GSE39582, COAD and TMA cohorts. (J) The distribution of metastatic or primary CRC tumors in GBP2 high and GBP2 low expression groups in the GSE131418 dataset. *P<0.05, ***p<0.001, ****p<0.0001 vs control group. COAD, colon adenocarcinoma; CRC, colorectal cancer; GBP2, guanylate binding protein 2; GO, gene ontology; MSI-H, high frequency microsatellite instability; MSI-L, low frequency microsatellite instability; MSS, microsatellite stability; ns, not significant; TMA, tissue microarray.

Next, we sought to discover the underlying mechanism differentiating IC and non-IC in pMMR/MSS CRC. It was reported that RNA-binding proteins (RBPs) can interact dynamically with other proteins, coding or non-coding RNA to affect the occurrence and development of a variety of malignant tumors.^20^ However, the relationship between RBPs and pMMR/MSS CRC has not yet been reported. Therefore, we calculated the DEGs between IC and non-IC in the COAD cohort to intersect with more than 3800 RBPs that have been reported,^21^ and 682 differentially expressed RBPs were obtained. Through univariate Cox proportional hazards regression analysis, 22 genes were finally obtained (figure 2A). The top five genes were shown in the figure 2B. We then calculated the correlation between the top five genes and the aforementioned immune signatures and found that S100 calcium binding protein B (S100B) and GBP2 were significantly positively correlated with these immune signatures (figure 2C). However, overexpression of S100B has been reported to promote CRC progression and can be used as an independent predictor of postoperative early relapse.^22^ Patients with high S100B expression also had a poorer clinical outcome.^22^ On the contrary, GBP2 is rarely reported among cancers and only Wang *et al* found that high GBP2 expression increased the sensitivity to paclitaxel in paclitaxel-resistant CRC.^12^ Therefore, GBP2 was selected for further study.

Based on the GEPIA2 database, we first found that GBP2 expression was significantly decreased in CRC compared with normal samples, and primarily decreased in the MSI-L/MSS CRC (figure 2D). The upregulation of GBP2 in the IC and MSI group was validated across the six independent CRC cohorts (figure 2E). In addition, Guinney *et al* proposed a robust classification system that can classify CRC into four CMS with distinctive features: CMS1 (MSI immune); CMS2 (canonical); CMS3 (metabolic) and CMS4 (mesenchymal).^23^ We found that the CMS1 subtype showed the highest GBP2 expression, whereas the CMS2 subtype showed the lowest GBP2 expression (figure 2F). Moreover, we analyzed somatic mutation data based on the COAD cohort to determine whether high and low GBP2 groups have different mutational profiles. A waterfall map showed that the top 30 genes showed a higher mutation rate in the high GBP2 group than in the low GBP2 group (online supplemental figure S4a). We further confirmed these data by applying two independent cohorts for validation. The results showed that the high GBP2 group was highly associated with the BRAF V600E mutation, while no significant differences were found in KRAS, TP53 and PIK3CA mutations (online supplemental figure S4b,c). We also found that upregulation of GBP2 was more likely to occur in the proximal (right) colon (online supplemental figure S4d). To further determine the potential biological functions of GBP2 in pMMR/MSS CRC, GSEA and gene ontology (GO) analysis were applied. Data in the six cohorts showed that patients with high GBP2 expression were significantly enriched in several immune-related pathways and functions, especially interferon-γ (IFN-γ) response, IFN-γ-mediated signaling pathways and T cell activation (figure 2G, H and online supplemental figures S5 and S6). Similar results were also obtained in MSI CRC (online supplemental figures S5 and S6). Taken together, these findings suggest that GBP2 participates in the immune response of pMMR/MSS CRC primarily by regulating the IFN-γ response and T cell activation.

### Low expression of GBP2 was associated with poor prognosis of CRC patients

Next, we examined the correlation between GBP2 and clinical characteristics. We first explored the prognostic role of GBP2 in CRC. The results showed that low expression of GBP2 predicts poor OS and relapse free survival in GSE39582, COAD and TMA cohorts (figure 2I). However, no relationship was found between GBP2 expression and prognosis in MSI CRC samples (online supplemental figure S7a). Moreover, in the COAD cohort, patients in the GBP2 low expression group were associated with worse N stage (p=0.036), M stage (p=0.017) and TNM stage (p=0.010) (online supplemental table S8). In the GSE41258 cohort, patients in the GBP2 low expression group were also associated with worse M stage (p=0.045) (online supplemental table S9). A high percentage of GBP2 positivity in the MSS patients of TMA cohort was also more likely correlated with low levels of T stage (p=0.0386) and N stage (p=0.0753) (online supplemental table S10). However, no significant differences were found in T stage (p=1), N stage (p=0.883), M stage (p=0.641) and TNM stage (p=0.452) in the GSE39582 cohort (online supplemental table S11). Considering the very disappointing data in stage IV CRC with ICB, we analyzed the relationship between GBP2 and metastasis in the GSE131418 dataset, which contains 878 primary CRCs and 257 metastatic CRCs.^24^ The results revealed that the number of metastatic patients were significantly higher in the GBP2 low expression group (35.1%, 199/567) compared with the high expression group (10.2%, 58/568) (figure 2J). Therefore, GBP2 may be closely related to CRC metastasis, but further studies on the correlation between GBP2 expression and clinical characteristics in CRC are needed.

10.1136/jitc-2021-004302.supp6 Supplementary data

10.1136/jitc-2021-004302.supp7 Supplementary data

10.1136/jitc-2021-004302.supp8 Supplementary data

10.1136/jitc-2021-004302.supp9 Supplementary data

### GBP2 expression positively correlates with tumor CD8+ T cell infiltration in pMMR/MSS CRC

**Figure 3:**
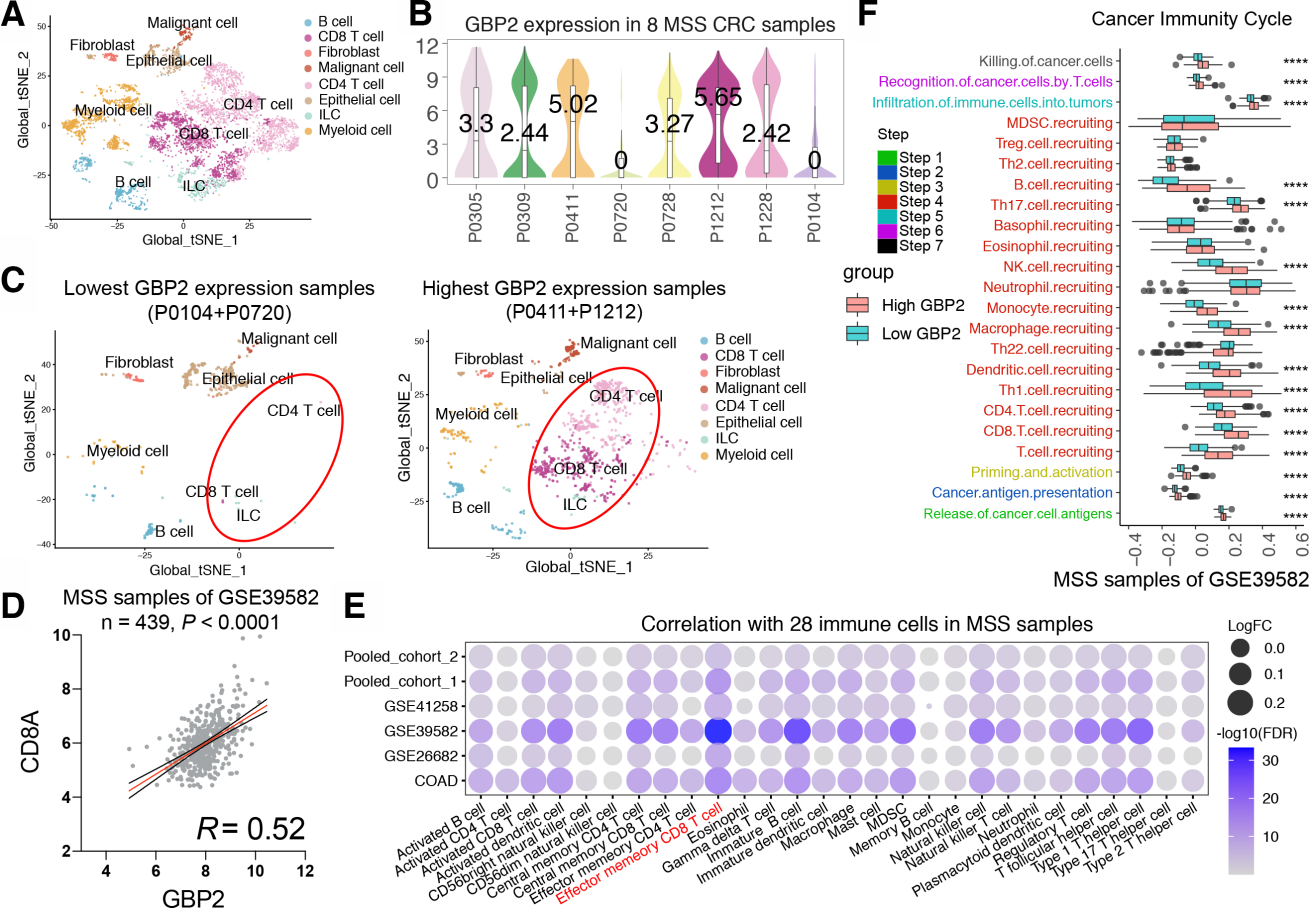
High GBP2 expression is associated with CD8 +T cell infiltration by bioinformatic approaches. (A) t-Distributed stochastic neighbor embedding (t-SNE) plot from eight MSS CRC patients. (B) Violin plot of GBP2 expression in eight MSS CRC patients. (C) t-SNE plot from two lowest GBP2 expression group (left) and highest GBP2 expression patients (right). (D) The correlation between GBP2 and CD8A in the MSS samples of GSE39582 cohort. (E) Bubble plot representation showing the correlation between GBP2 expression and 28 immune cells in the MSS samples of six cohort. (F) Differences in the various steps of the cancer immunity cycle between high-GBP2 and low-GBP2 groups in the MSS samples of GSE39582 cohort. ****P<0.0001 vs control group. CRC, colorectal cancer; GBP2, guanylate binding protein 2; MSS, microsatellite stability; FDA, Food and Drug Administration; MDSC, myeloid-derived suppressor cell.

To further explore the relationship between GBP2 and the immune microenvironment of MSS CRC, we analyzed single-cell RNA sequencing data from eight MSS CRC patients (figure 3A). We found a significant increase in CD4 +and CD8+T cell infiltration in the two patients with highest GBP2 expression (figure 3B, C). Furthermore, GBP2 was positively correlated with CD8A in the six cohorts (figure 3D; online supplemental figure S7b–g). We also used the ssGSEA algorithm to estimate the infiltration levels of 28 infiltrating immune cells in each MSS CRC sample. Likewise, we found that a great relevance between GBP2 expression and an inflamed immune microenvironment and was positively associated with the CD8 +T cells majorly across the six cohorts (figure 3E). Similar results were obtained in MSI CRC (online supplemental figure S8a). In addition, we found that GBP2 is highly associated with most of the antitumor immunity steps, such as release of cancer cell antigens (step 1), cancer antigen presentation (step 2), priming and activation (step 3), trafficking of immune cells to tumors (step 4) (mainly those that exert antitumor immunity). No significant differences were found in trafficking of myeloid-derived suppressor cell (MDSC) and Treg cells (figure 3F and online supplemental figure S9).

**Figure 4:**
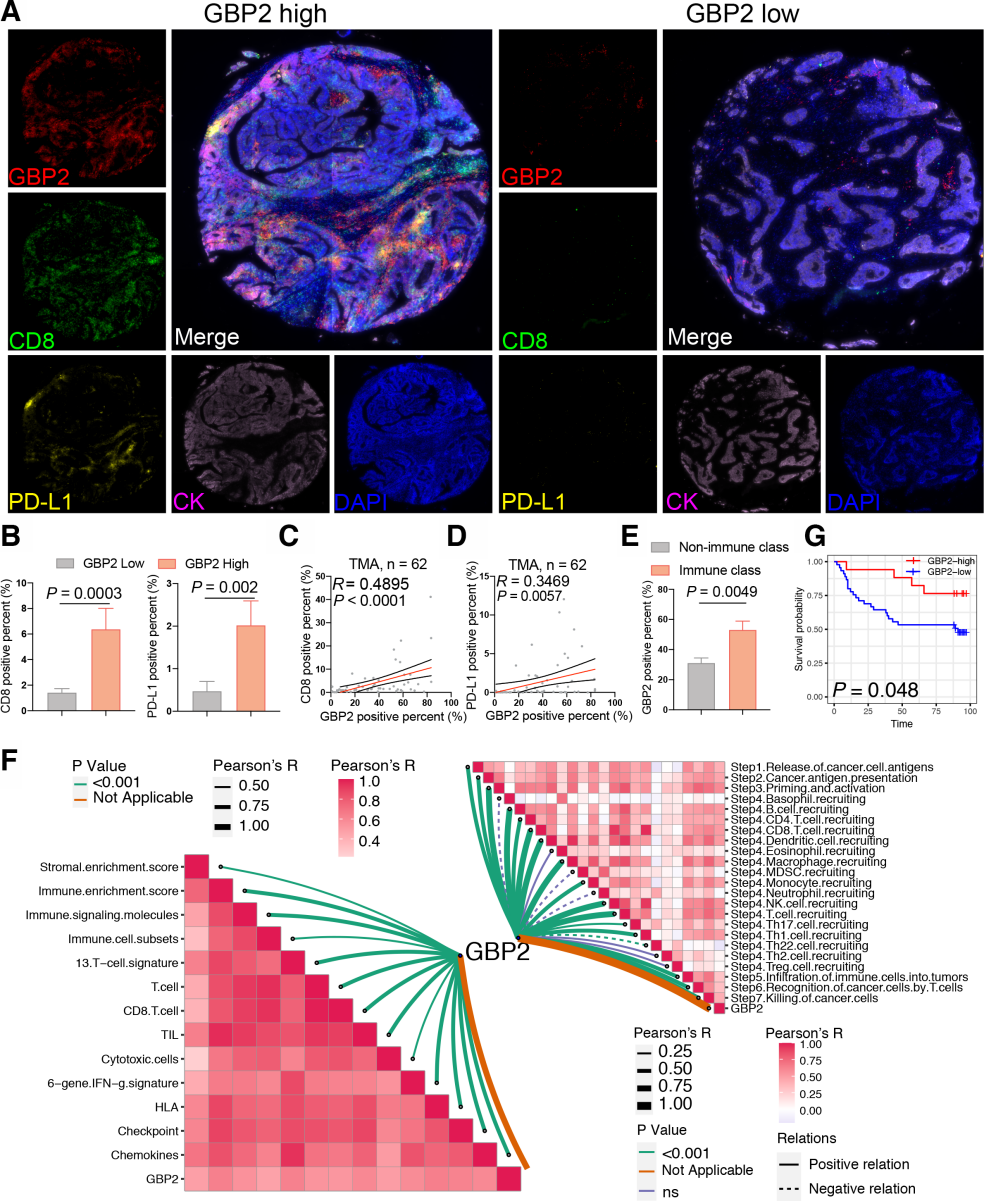
Verification of high GBP2 expression is associated with CD8 +T cell infiltration. (A) Expression of GBP2, CD8 and PD-L1 in the TMA cohort of 62 MSS CRC were detected using immunofluorescence. Representative costaining images of GBP2, PD-L1, and CD8 in the high and low GBP2 expression. Scale bars: 200 µm. (B) Box plots showing the positive percent of CD8 and PD-L1 between the high and low GBP2 expression group of the MSS samples of TMA cohort. (C) Correlation between the GBP2 positive percent and CD8 positive per cent detected using immunofluorescence. (D) Correlation between the GBP2 positive percent and PD-L1 positive per cent detected using immunofluorescence. (E) TMA were divided into two immune (>5%) and non-immune class (≤5%) based on the positive percent of CD8 +T cells. Box plots showing the positive percent of GBP2 between the immune and non-immune class of the MSS samples of TMA cohort. (F) (Bottom left) Correlations between GBP2 and 13 immune cell or immune response signatures. (Top right) Correlations between GBP2 and the steps of the cancer immunity cycle. (G) Survival analysis showing the relationship between GBP2 levels and the OS of patients in the MSS samples of TMA. CRC, colorectal cancer; GBP2, guanylate binding protein 2; MSS, microsatellite stability; OS, overall survival; TMA, tissue microarray.

Since the above conclusions were based on bioinformatic analysis, further validation was required to provide sufficient proof that high expression of GBP2 was associated with higher tumor CD8 +T cell infiltration in pMMR/MSS CRC. Thus, we validated these results in a TMA cohort. A total of 62 MSS CRC samples were included for subsequent analysis based on the results of nuclear immunohistochemical staining of four MMR proteins (online supplemental table S2). We found that samples with a high percentage GBP2-positivity showed a higher percentage positivity and cell counts of CD8 and PD-L1 (figure 4A, B and online supplemental figure S10a). In contrast, a lower percentage positivity and cell counts of CK were observed in these sample (online supplemental figure S10b). Consistently, GBP2 percentage positivity was positively correlated with CD8 and PD-L1 percentage positivity (figure 4C, D). We also divided MSS CRC samples in the TMA into IC and non-IC groups based on the percentage positivity of CD8 +T cells. The results further confirmed that the rate of GBP2 positivity was higher in the IC group compared with the non-IC group (figure 4E). Survival analysis showed that a high percentage of GBP2 positivity was associated with better OS in MSS CRC of TMA cohort (figure 4G). Taken together, these results indicate that GBP2 correlates with higher infiltration of CD8 +T cells in pMMR/MSS CRC (figure 4F).

### GBP2 expression positively correlates with the expression and secretion of CXCL10 and CXCL11 under IFN-γ stimulation in pMMR/MSS CRC

**Figure 5:**
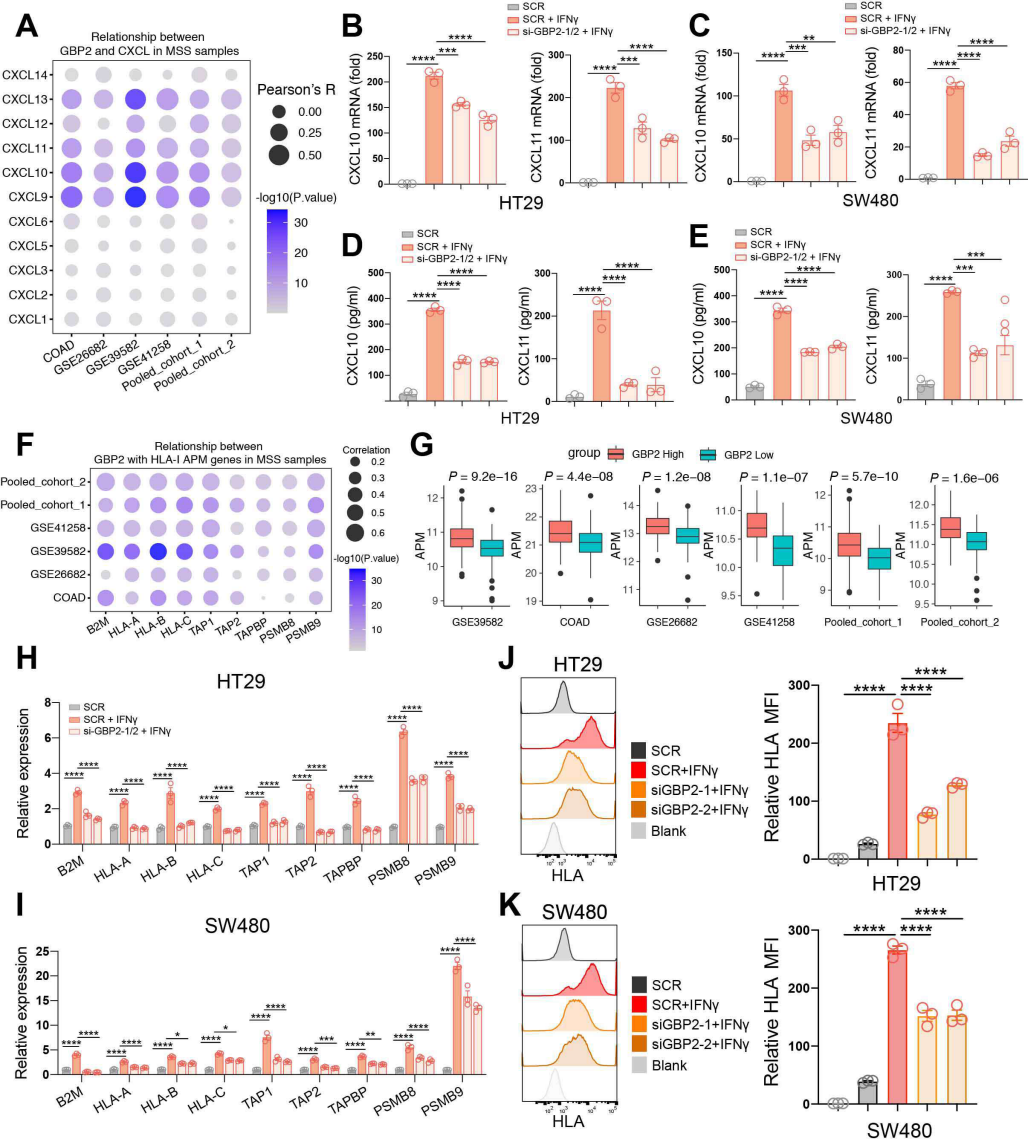
GBP2 expression positively correlates with higher expression and secretion of CXCL10/CXCL11 and the APM expression. (A) Bubble plot representation showing the correlation between GBP2 and CXCL genes in MSS samples of the six cohort. (B, C) HT29 and SW480 cells were transfected with scramble (SCR) and GBP2 siRNA for 48 hours, and then treated with 10 ng/mL IFN-γ for 24 hours. The CXCL10, CXCL11 expression were analyzed by RT- qPCR. (D, E) Culture supernatants of HT29 and SW480 cells were collected after 24 hours IFN-γ treatment, and the protein levels of CXCL10 and CXCL11 were measured using ELISA. (F) Bubble plot representation showing the correlation between GBP2 and HLA-I APM genes in MSS samples of the six cohort. (G) The APM score calculated by ssGSEA between high and low GBP2 expression group across all the six cohorts. (H, I) HT29 and SW480 cells were transfected with scramble (SCR) and GBP2 siRNA for 48 hours, and then treated with 4 ng/mL IFN-γ for 24 hours. The HLA-I APM genes expression were analyzed by RT-qPCR. (J, K) Typical histogram and quantification of median fluorescence intensity (MFI) of HLA FACS plot of blank, SCR, SCR +IFN-γ, si-GBP2-1/2+IFN-γ in HT29 and SW480 cells. *P<0.05, **p<0.01, ***p<0.001, ****p<0.0001 vs control group. APM, antigen processing and presentation machinery; COADM, colon adenocarcinoma; GBP2, GBP2; MSS, microsatellite stability.

Since the high correlation of GBP2 expression with higher CD8 +T cell infiltration, we analyzed the relationship between GBP2 expression and the CXCL family. CXCLs play an important role in the recruitment, migration, and activation of immune cells, especially CXCL9, CXCL10, and CXCL11.^25^ We revealed that GBP2 expression was markedly positively associated with CXCL9, CXCL10, CXCL11 and CXCL13 levels in the six cohorts (figure 5A; online supplemental figure S8b). Meanwhile, we found that IFN-γ mainly activated the expression of CXCL10 and CXCL11 in HT29 and SW480 cells (online supplemental figure S11a, b). Therefore, we primarily focused on studying the relationship between GBP2 and CXCL10/11. To verify the above findings, GBP2 expression was silenced using the small interfering RNA (siRNA) in the two MSS CRC cell lines (HT29 and SW480) (online supplemental figure S11c, d). The results showed that GBP2 itself had no effect on the expression of CXCL10/11 (online supplemental figure S12a, b). However, the mRNA levels of CXCL10/11 were significantly downregulated in GBP2-knockdown cells under IFN-γ stimulation (figure 5B, C). ELISA results further confirmed that downregulation of GBP2 could inhibit the protein expression of CXCL10 and CXCL11 in response to IFN-γ (figure 5D, E). Taken together, these results indicate that high GBP2 expression correlates with higher expression and secretion of CXCL10 and CXCL11.

### GBP2 expression positively correlates with the expression of HLA-I APM under IFN-γ stimulation in pMMR/MSS CRC

Cytotoxic CD8 +T cells can kill tumor cells by recognizing HLA class I (HLA-I) APM on the cancer cells. APM majorly includes antigen peptide generation (PSMB9, PSMB8), transport (TAP1, TAP2), loading (TAPBP) and presentation of the HLA-I complex (B2M-related HLA-A, HLA-B and HLA-C).^26^ Here, we found that GBP2 expression was significantly positively correlated with HLA-I APM expression in the six CRC cohorts (figure 5F; online supplemental figure S8c). Patients with high GBP2 expression not only had a higher level of CD8 +T cell infiltration, but also had a higher level of HLA-I APM expression in the six pMMR/MSS CRC cohorts (figure 5G). The results also showed that GBP2 itself had no effect on the expression of HLA-I APM (online supplemental figure S12c,d). However, HLA-I APM expression was significantly reduced in GBP2-knockdown cells in response to IFN-γ (figure 5H, I). Similarly, flow cytometry also found that knockdown of GBP2 suppressed the expression of MHC-I under IFN-γ stimulation (figure 5J, K). Taken together, these results indicate that high GBP2 expression correlates with higher expression HLA-I APM.

### Upregulation of GBP2 enhances the activation of signal transducer and transcription activator 1 phosphorylation by competing with SHP1 for binding to phosphorylated-signal transducer and transcription activator 1 in pMMR/MSS CRC

**Figure 6:**
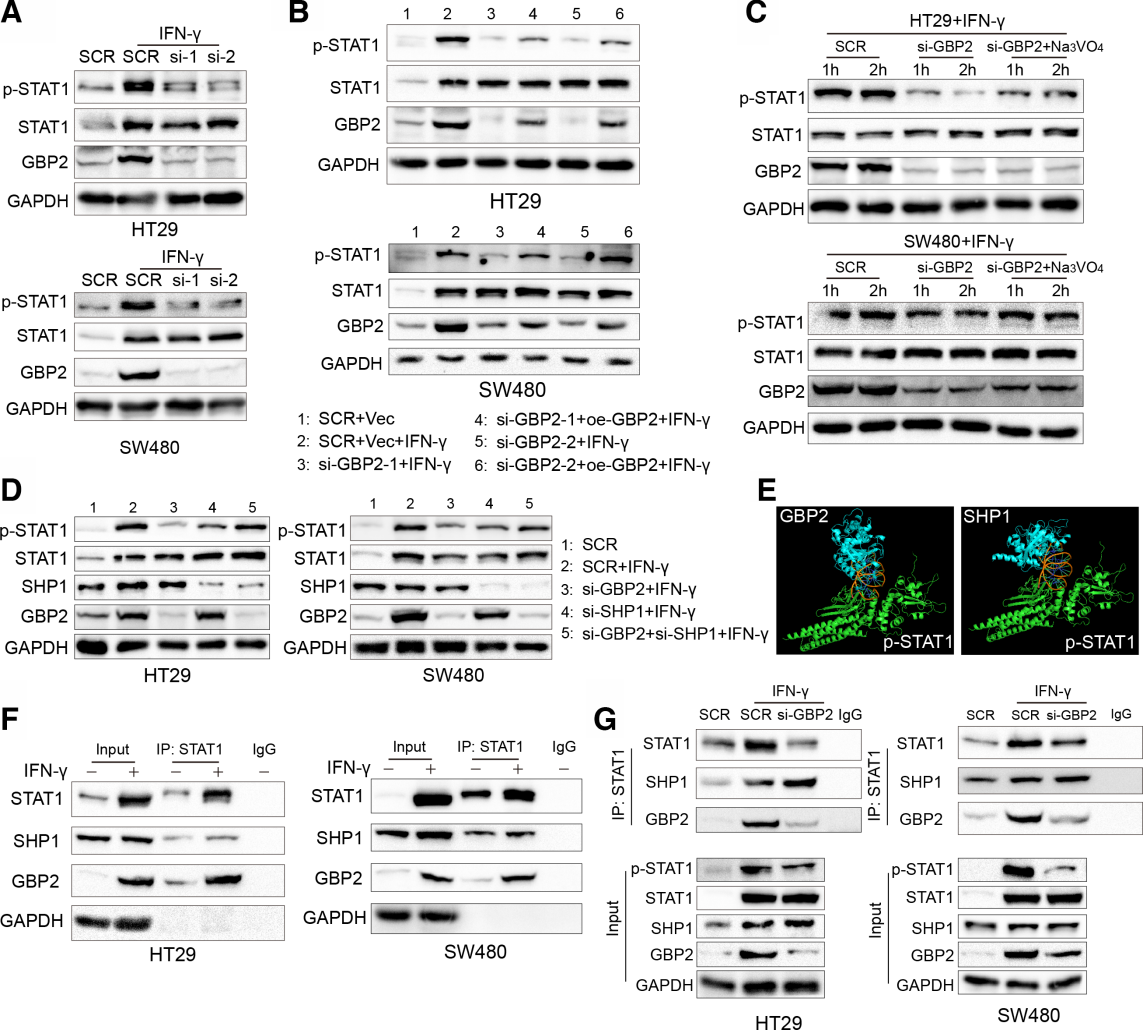
Diminished phosphorylation of STAT1 is mediated by the downregulation of GBP2. (A) HT29 and SW480 cells were transfected with scramble (SCR) and GBP2 siRNA for 48 hours, and then HT29 and SW480 were treated with 10 ng/mL IFN-γ for 12 hours and 24 hours, respectively, and cell lysates were collected and analyzed by western blot. (B) HT29 and SW480 cells were transfected with SCR, vector (Vec), GBP2 plasmid (oe-GBP2) and GBP2 siRNA for 36 hours, and then HT29 and SW480 were treated with 10 ng/mL IFN-γ for 12 hours and 24 hours, respectively, and cell lysates were collected and analyzed by western blot. (C) HT29 and SW480 cells were transfected with SCR and GBP2 siRNA for 36 hours, and then HT29 and SW480 were treated with 10 ng/mL IFN-γ for 12 hours and 24 hours, respectively. The si-GBP2 group were treated with 500 µm Na_3_VO_4_ for 1 and 2 hours, respectively. Cell lysates were collected and analyzed by western blot. (D) HT29 and SW480 cells were transfected with SCR, SHP1 and GBP2 siRNA for 36 hours, and then HT29 and SW480 were treated with 10 ng/mL IFN-γ for 12 hours and 24 hours, respectively, and cell lysates were collected and analyzed by western blot. (E) Prediction of the p-STAT1 and GBP2 (left) and p-STAT1 and SHP1 (right) interaction using Cluspro (https://cluspro.bu.edu/queue.php) and shown by PyMOL. (F) HT29 cells and SW480 cells were treated with 10 ng/mL IFN-γ for 24 hours. Then, cell lysates were used for immunoprecipitation with anti-STAT1 antibody and analyzed by western blot with the indicated antibodies. (G) HT29 cells and SW480 were transfected with SCR and GBP2-siRNA for 36 hours, and then treated PBS or IFN-γ (10 ng/mL) for 24 hours. Then, cell lysates were used for immunoprecipitation with anti-STAT1 antibody and analyzed by western blot with the indicated antibodies. GBP2, guanylate binding protein 2; STAT1, signal transducer and transcription activator 1; PBS, phosphate buffer saline.

Next, we sought to mechanistically illustrate the effect of GBP2 on the immune microenvironment of pMMR/MSS CRC. It has been reported that GBP2 is an interferon stimulus gene (ISG), and its expression can be significantly increased after IFN-γ treatment.^27^ Meanwhile, signal transducer and transcription activator 1 (STAT1) phosphorylation was significantly activated after IFN-γ stimuli.^28^ Therefore, we investigated whether the alteration of GBP2 expression could regulate the activation of STAT1 phosphorylation in pMMR/MSS CRC. We found that silencing of GBP2 reduced the activation of phosphorylated STAT1 (p-STAT1) in the HT29 and SW480 cells (figure 6A). Reconstruction of GBP2 in the GBP2-silenced cells can increase the activation of p-STAT1 (figure 6B).

In addition, STAT1 phosphorylation can be inhibited by directly interacting with negative regulators, such as SH2-containing protein tyrosine phosphatase 1 (SHP1).^29^ We found that the above reduced STAT1 phosphorylation can be rescued after treatment with Na_3_VO_4_, a non-specific tyrosine phosphatase inhibitor (figure 6C). Moreover, downregulation of SHP1 impaired STAT1 dephosphorylation in the GBP2-silenced cells (figure 6D). Our previous study also found that another ISG, retinoic acid inducible gene-I, can promote STAT1 phosphorylation by competing with SHP1 for binding to STAT1.^30^ Therefore, we hypothesized that GBP2 can also compete with SHP1 for binding to STAT1. We first docked the SHP1 protein (PDB id: 4GRY) and GBP2 protein (PDB id: 6VKJ) with the p-STAT1 protein (PDB id: 1BF5) using ClusPro 2.0.^31^ The results showed that GBP2 and SHP1 had similar interaction domains with STAT1 (figure 6E). To further validate this hypothesis, we conducted co-immunoprecipitation experiments and found that GBP2 and SHP1 interacting with STAT1 were significantly increased under IFN-γ stimulus in vitro (figure 6F). Furthermore, the interaction between SHP1 and p-STAT1 was increased in GBP2-silenced cells (figure 6G). Taken together, these results indicate that GBP2 enhances STAT1 phosphorylation by competing with SHP1 for binding to STAT1.

### GBP2 loss blunts the efficacy of PD-1 blockade in vivo

**Figure 7:**
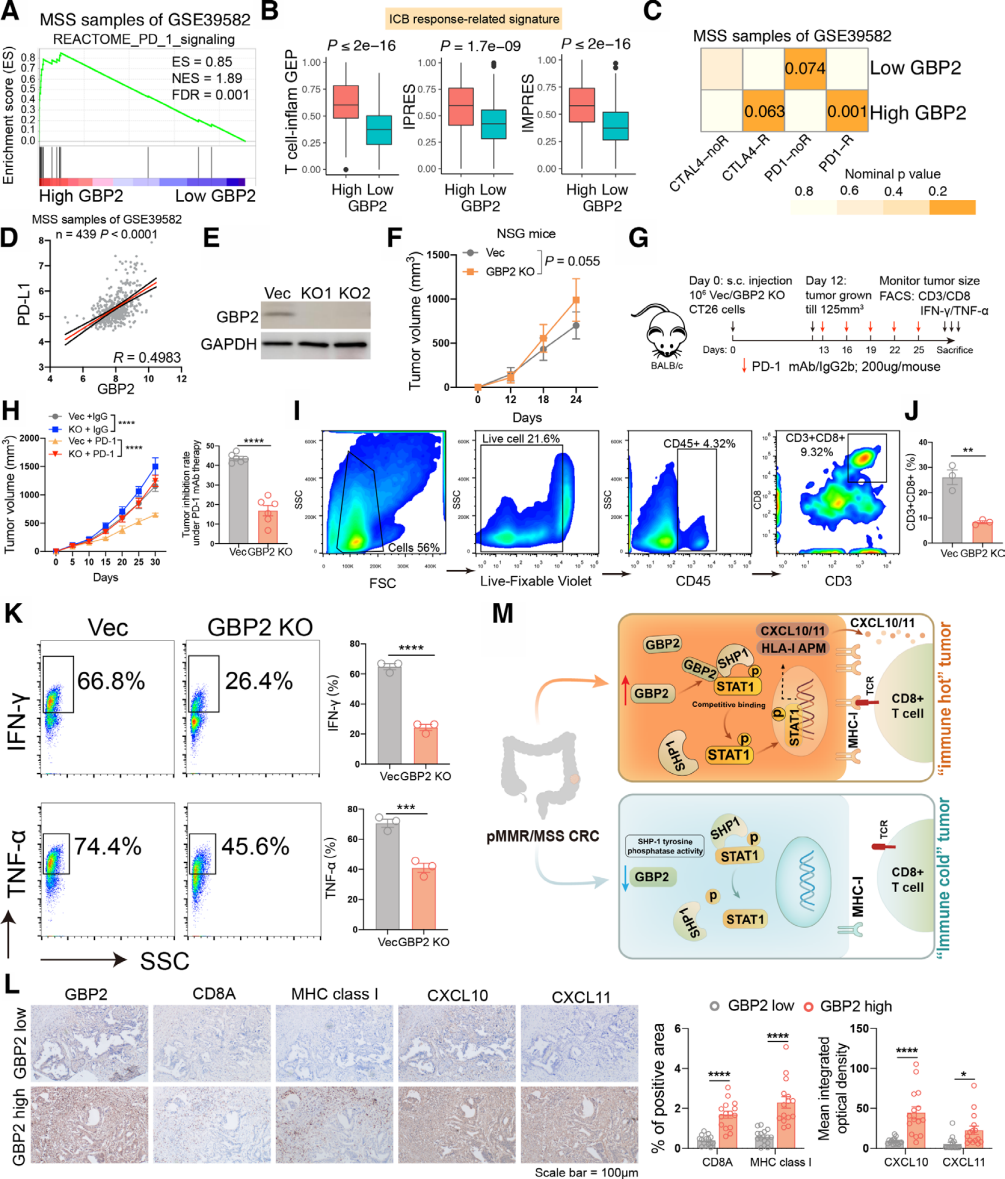
High expression of GBP2 is essential for responsiveness to PD-1 blockade in pMMR/MSS CRC. (A) GSEA plots of reactome PD-1 signaling and showing positively correlation with higher expression of GBP2 in the MSS samples of GSE39582. (B) Box plots showing expression of ICB response-related signatures between high and low GBP2 group in the GSE39582. (C) Submap analysis demonstrates that the high GBP2 group in the MSS samples of GS39582 cohort are nearly identical to the PD1-response (PD1-R) group defined in the melanoma cohort. (D) The correlation between GBP2 and PD-L1 in the MSS samples of GSE39582. (E) Transfection efficiency of two GBP2 knockout CT26 cells. (F) Time-course evaluation of NSG tumor volumes measured every 6 days. (G) Schematic diagram showing the grouping and treatment plan of the in vivo study: BALB/c mice were inoculated with 10^6^ Vec or GBP2 KO CT26 cells and received PD-1 mAb treatment or IgG2b control at the indicated time points. (H) (left) Time-course evaluation of BALB/c tumor volumes measured every 5 days; (right) Tumor inhibition rate for GBP2 KO cells relative to Vec cells under anti-PD-1 treatment, respectively. (I) Representative dot blot of flow cytometric analysis of CD45 +CD3+CD8+cells. (J) The quantitative percentage of CD8 between Vec and GBP2 knockout tumors were shown. (K) Representative images and statistical quantitation of the FACS analysis of the percentage of IFN-γ+CD8+ and TNF-α+CD8+TILs from Vec and GBP2 KO xenografts. (L) Representative photographs show the intratumoral expression of CD8A, MHC class I, CXCL10, and CXCL11 in samples with high GBP2 and in samples with low GBP2 in the same fields, on serial sections in CRC. Scale bars: 100 µm. The corresponding quantitative results were also shown. (M) A schematic show that the upregulation of GBP2 expression promotes the expression of CXCL10/11 and APM by competitively binding p-STAT1 with SHP1, thereby enhancing the anti-PD-1 response in the ‘immune hot’ MSS CRC. *P<0.05, **p<0.01, ***p<0.001, ****p<0.0001 vs control group. APM, antigen processing and presentation machinery; CRC, colorectal cancer; ES, enrichment score; FDR, false discovery rate; GBP2, guanylate binding protein 2; ICB, immune checkpoint blockade; MHC, immunohistochemistry; MSS, microsatellite stability; NES, normalized enrichment score; STAT1, signal transducer and transcription activator 1.

It was reported that p-STAT1 expression may be a potential biomarker for ICB in breast cancer patients.^32^ Therefore, we speculated that GBP2 may affect the efficacy of immunotherapy in pMMR/MSS CRC. We first found that patients with high GBP2 expression were highly positively correlated with reactome PD-1 signaling (figure 7A and online supplemental figure S13). Higher enrichment scores of T cell inflamed GEP, IPRES and IMPRES signatures were also observed in the high GBP2 group (figure 7B and online supplemental figure S14). We further analyzed the probability of responding to ICB through SubMap between the high- and low- GBP2 groups. By comparing our GBP2 expression profiles with a published melanoma immunotherapy dataset,^33^ the results showed that high GBP2 group were highly similar to the PD-1-response group in the six cohorts (figure 7C and online supplemental figure S15). A high correlation was also observed between GBP2 and PD-L1 in the six cohorts (figure 7D and online supplemental figure S16).

To test the hypothesis, we constructed two CT26 cell lines that lacked GBP2 (figure 7E). We found that only a slight increase in tumor volume was observed in GBP2 KO tumors implanted in immunodeficient NSG mice (figure 7F). Nevertheless, in wild-type mice, the tumor volume of GBP2 KO +IgG group was significantly larger than that for Vec+IgG group. Furthermore, anti-PD-1 treatment resulted in a tumor inhibition rate of about 43.7%, whereas depletion of GBP2 only caused about 16.9% tumor inhibition rate, indicating a potential role of GBP2 in the responsiveness to PD-1 blockade (figure 7G, H). A Transwell assay revealed that conditional medium (CM) obtained from IFN-γ-treated vec CT26 increased CD8 +T cell migration but had no effect on CM derived from IFN-γ-treated GBP2-KO CT26 culture. Antibody against CXCR3 pretreatment of T cells inhibited migration of T cells to the CM from IFN-γ-treated vec culture, but addition of recombinant murine CXCL10/11 to the CM from IFN-γ-treated GBP2-KO culture restored T cell migration (online supplemental figure S17). We also found that the tumors in the GBP2-KO group had lower levels of CD3 +CD8+T cell infiltration throughflow cytometry analysis (figure 7I, J). FACS data also indicated decreased IFN-γ and TNF-α levels in the GBP2-KO group (figure 7K). Finally, we determined the expression levels of CD8a, MHC class I and CXCL10/11 in GBP2-high and -low CRC tumors via immunohistochemistry (IHC). We found that intratumoral CD8a, MHC class I and CXCL10/11 expression also positively correlated with GBP2 levels in CRC tumors (figure 7L). Taken together, these results indicate that tumors expressing high levels of GBP2 are sensitized to anti-PD-1 therapy (figure 7M).

## Discussion

Due to the less inflamed immune microenvironment of pMMR/MSS CRC, the effect of monotherapy was ineffective. At present, new combinatorial regimens in pMMR/MSS CRC are being explored. In this study, we first categorized the pMMR/MSS CRC into immune and non-immune classes based on a total of 1424 patients in the six independent cohorts. GBP2 was selected and identified as a gene of interest due to its prognostic importance and high correlation with immune cells or immune response. We found that GBP2 expression was upregulated in the immune class and was strongly correlated with CD8 +T cell infiltration through bioinformatics algorithms and multiplex IHC. Furthermore, we confirmed that upregulation of GBP2 could increase the APM and CXCL10/11 expression on IFN-γ stimulus. Mechanistically, GBP2 promoted STAT1 phosphorylation by competing with SHP1 for binding to STAT1 in MSS CRC cells. Importantly, MSS patients with higher GBP2 expression were highly associated with a favorable response to anti-PD-1 therapy. Experiments in tumor-bearing mice further confirmed that GBP2 loss abrogated the efficacy of PD-1 blockade.

With the development of microarray technology, increasingly evidences shows that GEP can be used to explore more reliable molecular subtypes. For instance, MSI CRC was classified into two subtypes using a nonnegative matrix factorization algorithm.^34^ A subpopulation of pMMR/MSS patients showed increased CD8(+) TILs together with up-regulated IFN-γ via immunohistochemistry.^35^ A recent study further showed that MSS patients with or without chromosome 20q (Chr20q) amplification represented two subtypes of MSS CRC with distinct mutation profiles and immune cell infiltrations.^36^ However, there are still no studies to identify the molecular subtype of pMMR/MSS CRC. Deeper understanding of the immune landscape of pMMR/MSS CRC might lead to the development of new combinatorial strategies to overcome ICB resistance. Here, we classified the MSS samples based on the six cohorts. We revealed that pMMR/MSS could be classified into two robust classes with distinct immune features. The immune class exhibited significant enrichment of signatures identifying immune cells or immune response, such as immunoscore, CD8 +T cells, TIL, HLA, checkpoints and chemokines. The immunofluorescence results of our TMA cohort also revealed differences in the distribution of CD8 +T cells. Therefore, our findings confirm the existence of “hot” immune microenvironment in pMMR/MSS CRC and the relevance with better ICB response.

Currently, more alternative approaches are required to enhance the ICB response in pMMR/MSS CRC patients. For example, it was reported that blocking interleukin 17A (IL-17A) could improve the tumor response to anti-PD-1 immunotherapy in MSS CRC. The combination therapy of IL-17A and PD-1 antibodies significantly increased the CD8 +T cell population.^37^ Low-dose decitabine also enhanced the effect of PD-1 blockade in MSS CRC by re-modulating the tumor microenvironment.^38^ The lack of recruitment of immune cells (mainly CD8 +T cells) to the tumor seems to be the fundamental obstacle to ICB efficacy. Therefore, based on the constructed immune and non-immune classes in MSS CRC, our study screened GBP2 as the gene of interest. There are few studies on the relationship between GBP2 and tumor progression. Yu *et al* indicated that GBP2 could enhance the glioblastoma invasion through Stat3/fibronectin pathway.^39^ In pancreatic adenocarcinoma (PAAD), GBP2 expression was significantly upregulated in PAAD tissues. The overexpression of GBP2 was highly associated with an advanced T stage and poor OS.^40^ Nevertheless, it was reported that higher expression of GBP2 was correlated with a favorable prognosis in breast cancer.^41^ Zhang *et al* further demonstrated that GBP2 inhibited breast cancer cell invasion and dynamin-related protein 1 (Drp1)-dependent mitochondrial fission by directly binding to Drp1.^42^ Only one study found that GBP2 acted as a tumor suppressor and could increase the sensitivity of paclitaxel-resistant CRC cells to paclitaxel through WNT signaling.^12^ Here, we found that low expression of GBP2 correlated with metastasis as well as poor prognosis in CRC. Collectively, these data suggest that GBP2 is a potential therapeutic target in CRC.

To further illustrate the critical role of GBP2, we performed GSEA and GO analysis. The results showed the IFN-γ pathway was the most relevant function. The premise of ICB is that T cells specific for cancer antigens recognize their targets on cancer cells and produce IFN-γ. Therefore, lack of IFN-γ signaling is one of the mechanisms of ICB resistance.^43^ For instance, Gao *et al* reported that patients identified as non-responders to anti-CTLA-4 (ipilimumab) had an average of 15.33 mutations in IFN-γ pathway genes as compared with an average of only one mutation in responders.^44^ The activation of IFN-γ and downstream expression of ISGs can predict response to immunotherapies in preclinical and clinical studies.^45 46^ In a melanoma patient cohort treated with ICB, all no-respondents with active CD8 +T cell signatures carried defects in IFN-γ pathway.^47^ These data highlight that loss of the IFN-γ signaling pathway is associated with primary resistance to ICB. As an ISG, GBP2 expression increases significantly after IFN-γ treatment, but the role of GBP2 in the IFN-γ response in MSS CRC remains unclear. Here, we found that GBP2 promoted STAT1 phosphorylation by competing with SHP1 for binding to STAT1 in MSS CRC. STAT1 has been reported to be a favorable prognostic biomarker in CRC.^48^ Meanwhile, high levels of CD8 +T cells were found in a subpopulation of pMMR CRC patients that were positive for PD-L1 and p-STAT1.^35^ Our TMA cohort also showed a higher PD-L1 positivity rate in the high GBP2 expression group. Thus, we suggest that GBP2 as a biomarker for enhancing the efficacy of and patient response to ICB therapy. However, the correlations between GBP2 and immunosurveillance and immunotherapy for pMMR/MSS CRC have not been reported.

Tumor immunosurveillance is comprised of ‘three Es phases’: elimination, equilibrium, and escape.^49^ When the tumors are clinically detectable, this indicates that they have progressed to the escape phase. Escape includes several complex processes, such as loss of APM, sensitivity to immune effector molecules (such as chemokine CXCL9/10/11), induction of T cell apoptosis and anergy. The induction of regulatory T cells also promotes tumor immune escape.^50^ Godoy *et al* first reported that high expression of GBP2 was correlated with the T-cell signature in breast cancer.^41^ Our single-cell and bulk RNA sequencing data demonstrated that GBP2 was highly associated with T-cell activation and CD8 +T cell infiltration. We validated this finding through immunofluorescence of our TMA cohort. Furthermore, the increased infiltration of CD8 +T cells may be attributed to the upregulation of CXCL10/11 promoted by GBP2. We further confirmed that GBP2 KO reduced the CD8 +T cell migration, whereas supplementation with CXCL10/11 restored T cell migration. Additionally, our data showed that GBP2 could enhance the APM expression, which was consistent with the study of Li *et al*. They first identified a long noncoding RNAs, LIMIT, which augments MHC-I expression and enhances antitumor immunity by activating the GBP/heat shock factor-1 axis. They also found that silencing GBP2 could reduce the MHC-I expression and abrogated the efficacy of PD-L1 blockade.^51^ Similarly, our study demonstrated that GBP2 was correlated with the PD-1/PD-L1 axis and mice bearing GBP2 KO CT26 cells showed reduced efficacy of PD-1 blockade. These data suggest that deletion of GBP2 can promote immune escape by inhibiting the expression of APM and CXCL10/11 and reduce the infiltration of CD8 T cells, thereby blunting the efficacy of ICB.

In summary, our study stratified the pMMR/MSS CRC into immune and non-immune classes and identified that GBP2 is a promising target for combinatorial therapy with ICB. We also revealed a novel mechanism by which GBP2 promotes STAT1 phosphorylation in pMMR/MSS CRC for the first time. Our work deepens our understanding of the immune microenvironment and could help provide precision immunotherapy for pMMR/MSS CRC.

10.1136/jitc-2021-004302.supp10 Supplementary data

10.1136/jitc-2021-004302.supp11 Supplementary data

10.1136/jitc-2021-004302.supp12 Supplementary data

## Data Availability

Data are available in a public, open access repository. All data relevant to the study are included in the article or uploaded as online supplemental information. The public datasets used and/or analyzed during the current study are available in the GEO database (https://www.ncbi.nlm.nih.gov/geo/) and TCGA database. All data relevant to the study are included in the article or uploaded as supplemental information.
