# Supplementary material for: Subtyping of microsatellite stability colorectal cancer reveals guanylate binding protein 2 (GBP2) as a potential immunotherapeutic target
Source: J Immunother Cancer. 2022 Apr 5;10(4):e004302. doi: 10.1136/jitc-2021-004302 (PMC8984016; doi:10.1136/jitc-2021-004302)
Supplement: Supplementary data [file jitc-2021-004302supp001.pdf]

2. METHODS and MATERIALS

2.6 Immune-related bioinformatic analysis

The relative abundance of 28 immune cell infiltration in each pMMR/MSS CRC sample was quantified via ssGSEA (**Table S4**) [1]. We used the "limma" R package to screen the differentially infiltrating cells in the tumors separated by median GBP2 expression (high versus low) [2]. The body activates the anti-tumor immune response to kill tumor cells, in which a series of steps must be initiated and allow to proceed repeatedly. Thus, Chen *et al* summarized these steps as the cancer-immunity cycle, involving the release of cancer cell antigens (Step 1), cancer antigen presentation (Step 2), priming and activation (Step 3), trafficking of immune cells to tumors (Step 4), infiltration of immune cells into tumors (Step 5), recognition of cancer cells by T cells (Step 6), and killing of cancer cells (Step 7) [3, 4]. The fate of tumor cells is determined by the levels of these steps. We evaluated the relative activities of the immunity cycle using ssGSEA (**Table S5**).

An unsupervised subclass mapping method (SubMap) (GenePattern module 'SubMap') (https://cloud.genepattern.org) was used to identify common subgroups between independent cohorts despite their technical differences [5]. A melanoma dataset including anti-PD-1 and anti-CTLA4 therapy was applied to measure the similarity with high- and low-risk GBP2 groups in the CRC datasets included in this study [6]. The single-cell transcriptome analysis for the eight MSS CRC patients (Smart-Seq2) was performed at http://crcleukocyte.cancer-pku.cn/ [7].

2.7 Gene set enrichment analysis (GSEA) and Gene Ontology (GO) functional annotation

GSEA was used to enrich the significant gene sets between the high and low GBP2 groups. The gene sets of "H.all.v7.4" were downloaded from the Molecular

Signatures Database and used as the reference set to represent well-defined biological states or processes. A false discovery rate (FDR) of < 0.05, |enrichment score (ES)| > 0.6 and normalized ES (NES) > 2 were used as the cutoff criteria. Additionally, Gene Ontology (GO) functional enrichment analysis for the top 200 differentially expressed genes (DEGs) calculated by "limma" was performed using the "clusterProfiler" R package [8].

2.8 Plasmids and siRNA transfection

Human pCMV3-GBP2 plasmid was purchased from Sinobiological. HT29 and SW480 cells were transfected with pCMV3-GBP2 plasmid using Lipofectamine 2000 (Invitrogen, USA) to overexpress GBP2. Vector pCMV plasmid was used as a control.

GBP2 and SHP1 siRNAs were purchased from Ribobio (Guangzhou, China). HT29 and SW480 cells were transfected with si-GBP2-1 and si-GBP2-2 using Lipofectamine 2000 (Invitrogen, USA) to knockdown GBP2 expression. Scramble siRNA was used as a control. Sequences of siRNAs were as follows: si-GBP2-1: GCTGAACCCTGATTTCATA, si-GBP2-2: GGAGCTTTCGCTAAAGCTA. si-SHP1: GCAAGAACCGCTACAAGAA.

2.9 Multiplex immunohistochemistry (mIHC) and immunohistochemistry

The CRC tissue microarray (TMA) (HColA180Su18) was purchased from the Shanghai Outdo Biotech. All the paraffin-embedded CRC specimens were obtained from the Biobank of the National Engineering Center for Biochip in Shanghai (the National Human Genetic Resources Sharing Service Platform, No. 2005DKA21300). The mIHC was performed using an Opal 7-color fluorescent IHC kit (NEL801001KT PerkinElmer). In brief, the concentration of the indicated antibodies was first optimized. A series of xylene-to-alcohol washes were performed to dewax and rehydrate using an automatic dewaxing dyeing machine (LEICAST5020, LEICA). Then, after heat-

induced antigen retrieval and removal of endogenous peroxidase, the slides were blocked and then incubated with the following primary antibodies: GBP2 (11854-1-AP, Proteintech), CD8 (PA067, Abcarta), PD-L1 (PA167, Abcarta) and cytokeratin (CK) (PA125, Abcarta). The secondary antibody was added to the slides and incubated at room temperature. After incubation with the opal dye (1:100), the antigen thermal retrieval was performed again. The above process was repeated to stain different primary antibodies. Finally, DAPI staining was performed. Fluorescence images were acquired using the TissueFAXS Viewer software (TissueGnostics). Vectra Polaris software (PerkinElmer) was used to automate the quantitative analysis of the expression level of GBP2, CD8, CK and PD-L1.

All MSS CRC samples were acquired from Zhongnan Hospital of Wuhan University. Informed consent was given by patients for all samples. Standard immunohistochemistry protocol was performed using the anti-GBP2 antibody (11854-1-AP, Proteintech), anti-MHC-I antibody (R27458; ZEN Bio), anti-CXCL10 antibody (10937-1-AP; Proteintech), anti-CXCL11 antibody (10707-1-AP; Proteintech) and anti-CD8A antibody (ab217344, Abcam). 5 fields from each sample were randomly selected for quantitative analysis.

2.10 Quantitative real-time PCR (qPCR)

Total RNA was extracted from HT29 and SW480 cells using Trizol (Invitrogen, USA) and converted to cDNA using a ReverTra Ace qPCR RT Kit (TOYOBO, Japan). Then, qPCR was performed using UltraSYBR mixture (Cwbio, China) on a Roche LightCycler 96 PCR system. Relative gene-expression quantification was quantified using the comparative CT method against GAPDH. The primer sequences are shown in **Table S6**.

2.11 Western blotting

Total protein fractions of CRC cells were obtained using NP40 lysis buffer (Beyotime, China) and their concentrations were determined using the BCA kits (Beyotime, China). Then, cell lysates (30 μg) were separated by 7.5-12% SDS-polyacrylamide gel electrophoresis and then transferred onto polyvinylidene fluoride (PVDF) membranes (Millipore, USA). After blocking with 5% fat-free milk, the membranes were incubated with specific anti-GBP2 (11854-1-AP, Proteintech, USA), anti-p-STAT1 (R25797, ZEN BIO, China), anti-STAT1 (385801, ZEN BIO, China), anti-SHP1 (201197, ZEN BIO, China), or anti-GAPDH (GB13002, Servicebio, China) overnight at 4 °C. Finally, enhanced chemiluminescence reagents (Thermo, USA) were applied to visualize the proteins after incubation with the secondary antibodies (GB23303, Servicebio, China).

2.12. Coimmunoprecipitation (CoIP)

Total protein fractions were collected for CoIP with anti-STAT1 antibody according to the manufacturer's protocol using an IP/CoIP kit (Absin, China). In brief, HT29 and SW480 cells were lysed with IP buffer (P0013J, Beyotime, China) containing protease and phosphatase inhibitors for 45 minutes at 4°C. The cell lysates were precleared with protein A/G-agarose beads for 1 hour and then incubated with IgG, anti-STAT1, overnight at 4°C with continuous inversion. The cell lysates were precipitated with protein A/G-agarose beads for 4 hours and then the immunoprecipitated beads were washed five times the following day. Bound proteins were eluted and subjected to immunoblot analysis with indicated antibodies.

2.13. Stable knockout (KO) cell line construction

The CRISPR/Cas9 based on pX459 plasmid was constructed. The guide RNA sequence was as follows: SGGFP: 5′-GGGCGAGGAGCTGTTCACCG-3′; mGBP2-SGRNA1: 5′- UGCUUUUCAGGCUUUUCCCU -3′; mGBP2-SGRNA2: 5'-

GUGUGUGCCUCACCCCAAGA -3′. For CRISPR/Cas9 plasmid transfection, CT26 cells were transfected according to instructions for the Lipofectamine 2000 and sorted by puromycin to acquire a monoclonal cell line. Finally, the GBP2 KO cells were identified via western blotting and cultured for further analysis.

2.14. Isolation of tumor infiltrating lymphocytes (TILs) and flow cytometry

CT26 tumor samples were prepared via mechanical separation and treated with collagenase P (2 mg/ml, Sigma) and DNase I (50 μg/ml, Sigma) for 15 min at 37 °C. The LIVE/DEAD Fixable Violet Dead Cell Stain Kit (#L34955; Thermo) was used to exclude dead cells. For surface marker analysis, live cells were re-suspended in 1 × PBS and stained with anti-mouse CD3 (17A2, 100204, Biolegend), CD4 (GK1.5, 100406, Biolegend), CD8a (53-6.7, 100708. Biolegend), or CD45 (30-F11, 103106. Biolegend). Intranuclear staining was performed using the Fixation and Permeabilization Solution (554722; BD Bioscience) according to the manufacturer’s instructions and APC anti-mouse IFN-γ (XMG1.2, Biolegend, 505808) and TNF-α (MP6-XT22, Biolegend, 506306) were used. When indicated, cytokine production was measured following 4 hours of *in vitro* stimulation, and Leukocyte activation cocktail (BD, 550583) was added to prevent excretion of the cytokines. In addition, HT29 and SW480 cells were dissociated and then incubated with APC HLA-A, B, C antibody (W6/32, Biolegend, 311409,) for 30 min at 4 °C. All stained cells were analyzed on an Attune NxT systems and the data was analyzed with FlowJo version 10 software.

2.15. Enzyme-linked immunosorbent assay (ELISA)

The culture supernatants of HT29 and SW480 cells were used to quantify the protein levels of CXCL10 and CXCL11 using ELISA kits (EK0735 and EK0737, Boster, China) according to the manufacturer’s instructions.

References:

[1] P. Charoentong, F. Finotello, M. Angelova, C. Mayer, M. Efremova, D. Rieder, H. Hackl, Z. Trajanoski, Pan-cancer Immunogenomic Analyses Reveal Genotype-Immunophenotype Relationships and Predictors of Response to Checkpoint Blockade, Cell reports 18(1) (2017) 248-262.
[2] M.E. Ritchie, B. Phipson, D. Wu, Y. Hu, C.W. Law, W. Shi, G.K. Smyth, limma powers differential expression analyses for RNA-sequencing and microarray studies, Nucleic acids research 43(7) (2015) e47.
[3] D.S. Chen, I. Mellman, Oncology meets immunology: the cancer-immunity cycle, Immunity 39(1) (2013) 1-10.
[4] L. Xu, C. Deng, B. Pang, X. Zhang, W. Liu, G. Liao, H. Yuan, P. Cheng, F. Li, Z. Long, M. Yan, T. Zhao, Y. Xiao, X. Li, TIP: A Web Server for Resolving Tumor Immunophenotype Profiling, Cancer research 78(23) (2018) 6575-6580.
[5] Y. Hoshida, J.P. Brunet, P. Tamayo, T.R. Golub, J.P. Mesirov, Subclass mapping: identifying common subtypes in independent disease data sets, PloS one 2(11) (2007) e1195.
[6] W. Roh, P.L. Chen, A. Reuben, C.N. Spencer, P.A. Prieto, J.P. Miller, V. Gopalakrishnan, F. Wang, Z.A. Cooper, S.M. Reddy, C. Gumbs, L. Little, Q. Chang, W.S. Chen, K. Wani, M.P. De Macedo, E. Chen, J.L. Austin-Breneman, H. Jiang, J. Roszik, M.T. Tetzlaff, M.A. Davies, J.E. Gershenwald, H. Tawbi, A.J. Lazar, P. Hwu, W.J. Hwu, A. Diab, I.C. Glitza, S.P. Patel, S.E. Woodman, R.N. Amaria, V.G. Prieto, J. Hu, P. Sharma, J.P. Allison, L. Chin, J. Zhang, J.A. Wargo, P.A. Futreal, Integrated molecular analysis of tumor biopsies on sequential CTLA-4 and PD-1 blockade reveals markers of response and resistance, Science translational medicine 9(379) (2017).
[7] L. Zhang, Z. Li, K.M. Skrzypczynska, Q. Fang, W. Zhang, S.A. O'Brien, Y. He, L. Wang, Q. Zhang, A. Kim, R. Gao, J. Orf, T. Wang, D. Sawant, J. Kang, D. Bhatt, D. Lu, C.M. Li, A.S. Rapaport, K. Perez, Y. Ye, S. Wang, X. Hu, X. Ren, W. Ouyang, Z. Shen, J.G. Egen, Z. Zhang, X. Yu, Single-Cell Analyses Inform Mechanisms of Myeloid-Targeted Therapies in Colon Cancer, Cell 181(2) (2020) 442-459.e29.
[8] G. Yu, L.G. Wang, Y. Han, Q.Y. He, clusterProfiler: an R package for comparing biological themes among gene clusters, Omics : a journal of integrative biology 16(5) (2012) 284-7.

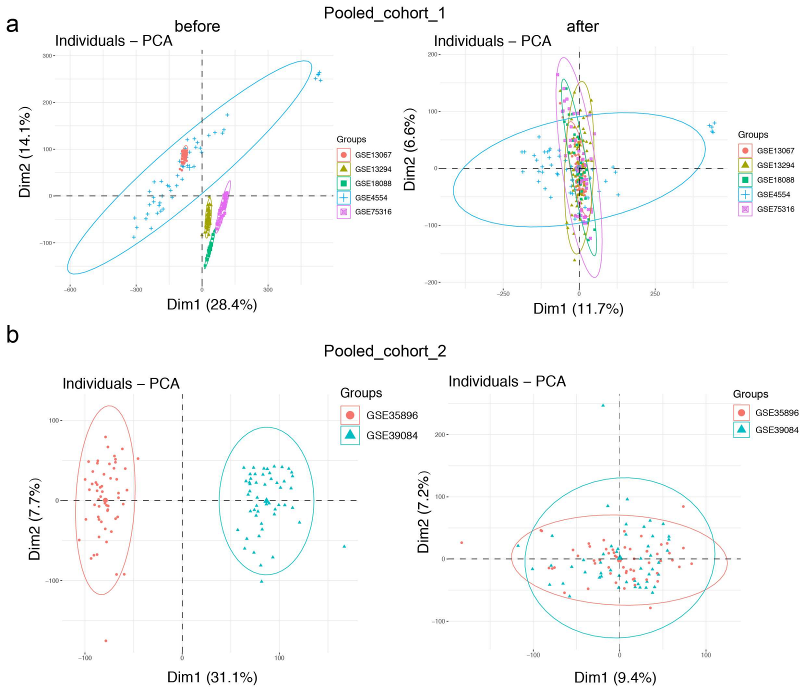

Figure S1: PCA showed the batch effect of pooled cohort 1 and 2 before and after the combination.

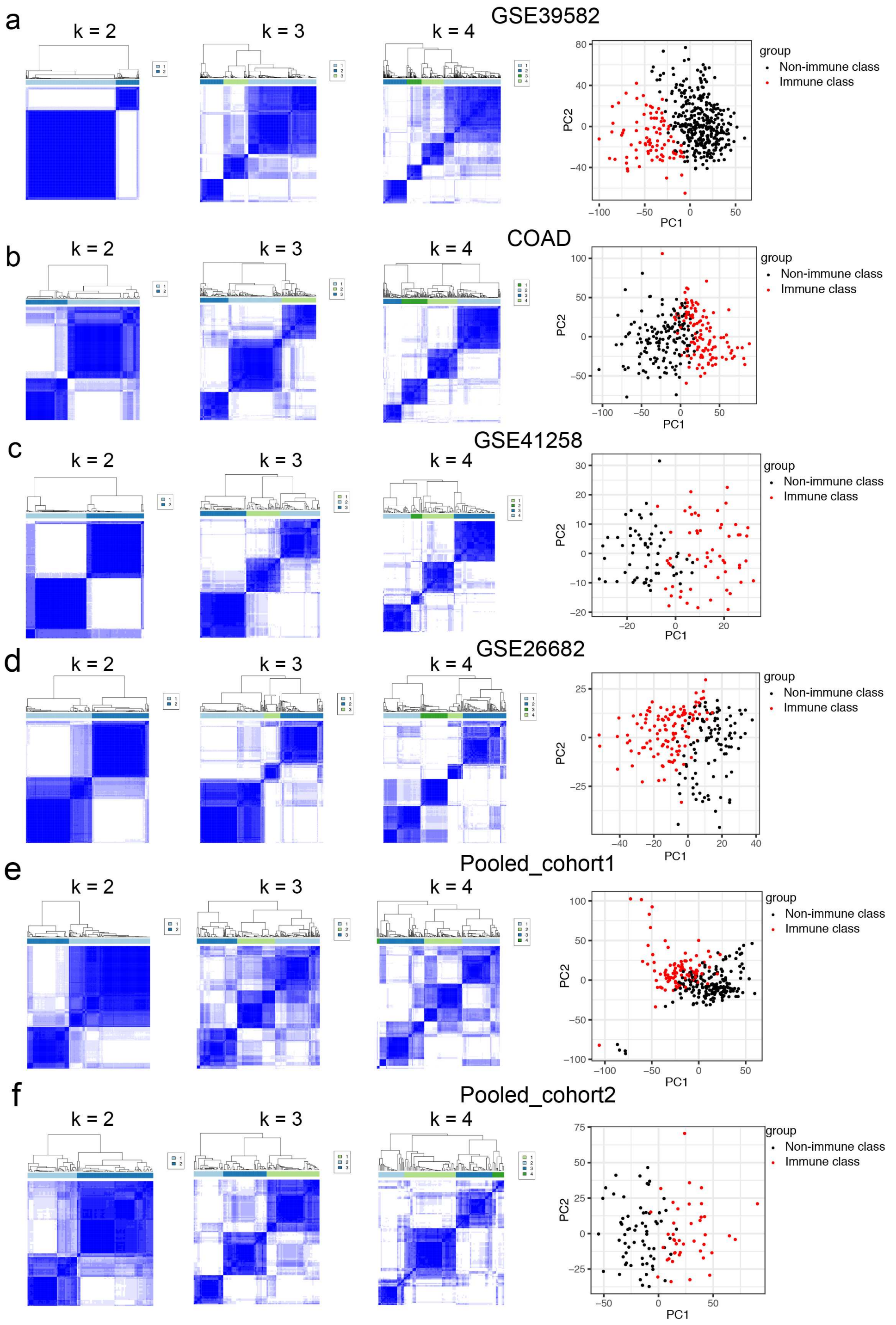

Figure S2: (a-f) Consensus matrices of the six cohort for k = 2 - 4. PCA results were showed in the right panel between immune and non-immune class.

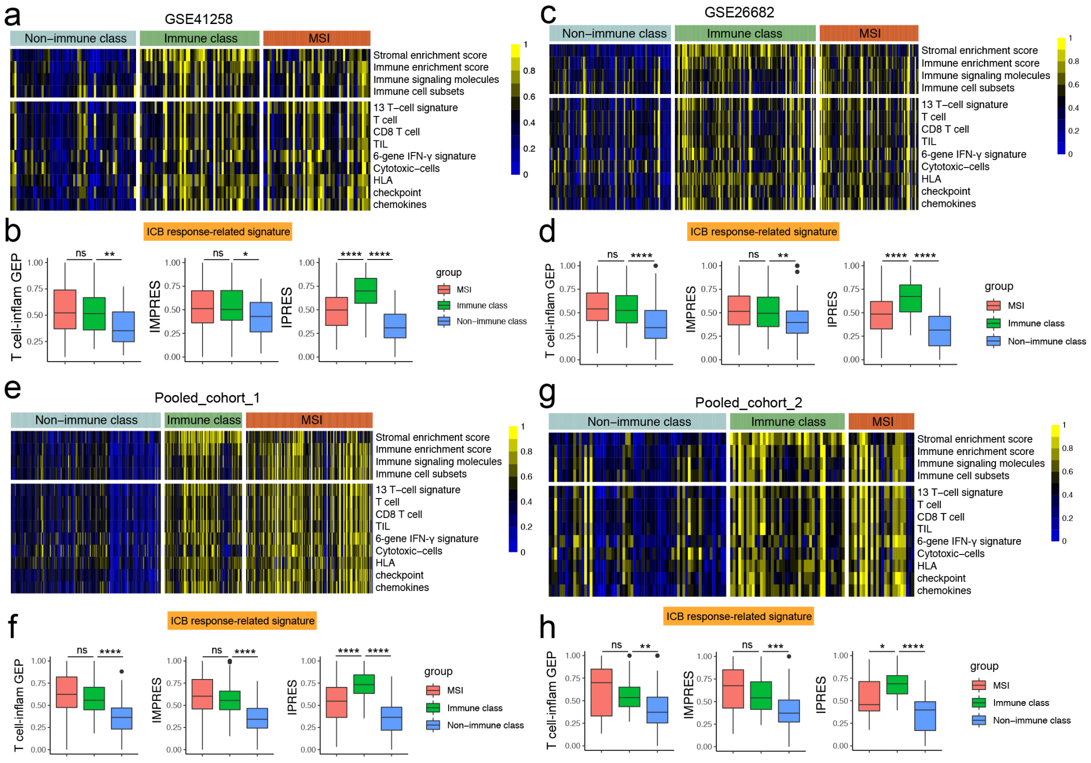

Figure S3: An immune class and a non-immune class were identified using consensus clustering in the **(a)** GSE41258, **(c)** GSE26682, **(e)** pooled_cohort_1 and **(g)** pooled_cohort_2 cohorts. In the heatmap, high and low ssGSEA scores of 13 immune cell or immune response signatures are represented in yellow and blue, respectively. **(b, d, f, h)** Box plots showing expression of ICB response-related signatures between immune class, non-immune class and MSI samples. ssGSEA, single-sample gene-set enrichment analysis; ICB, immune checkpoint blockade; CRC, colorectal cancer; pMMR/MSS, proficient-mismatch-repair/microsatellite stability; MSI, microsatellite instability.

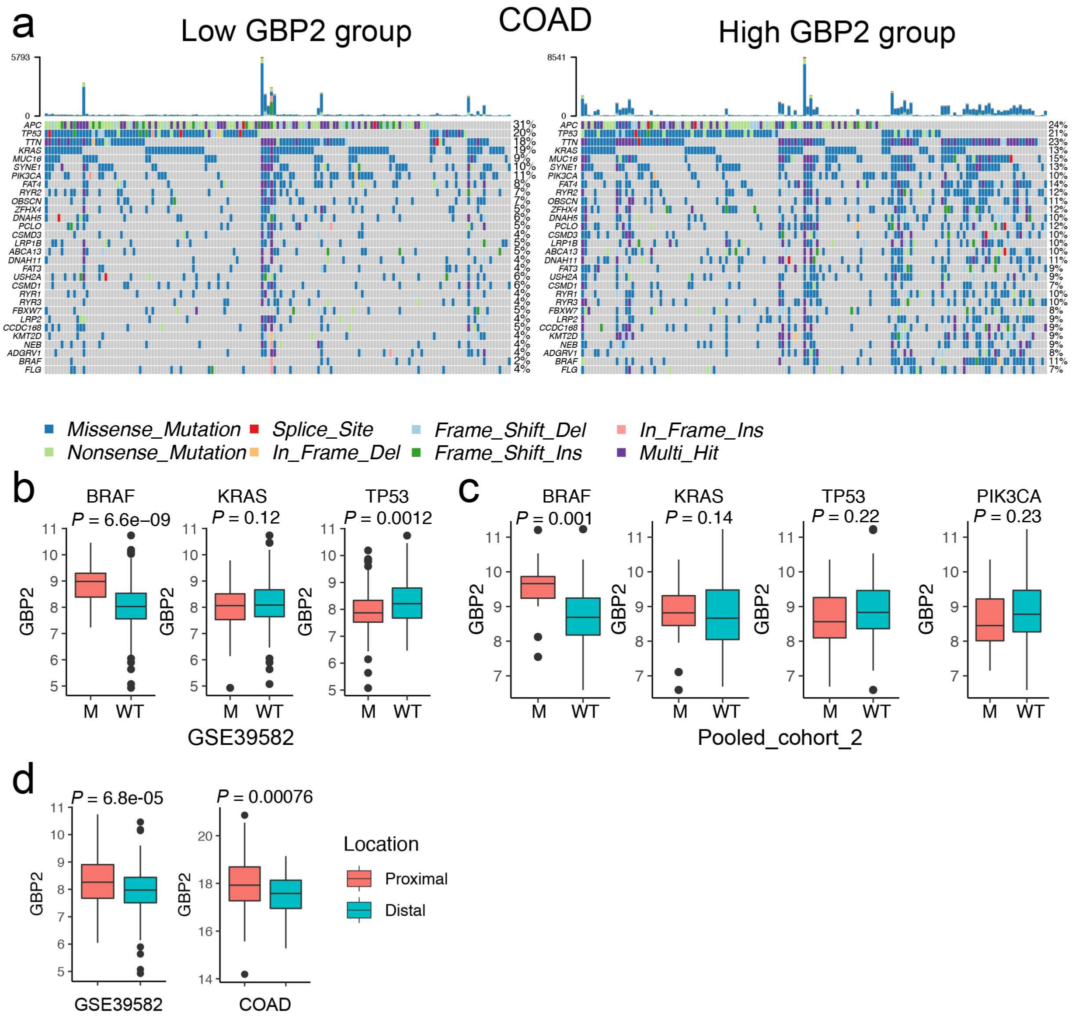

Figure S4: **(a)** The waterfall plots showed top 30 gene alterations in the GBP2 high and low expression group, respectively. **(b, c)** Box plots showing expression of GBP2 between BRAF, KRAS, TP53 and PIK3CA mutations (M) in the GSE39582 and Pooled cohort 2 cohorts. **(d)** Box plots showing expression of GBP2 between proximal and distal location in the GSE39582 and COAD cohorts.

a

MSI samples of COAD

Gene sets	NES	ES	FDR
Allograft rejection	2.25	0.71	<0.001
IFNγ response	2.16	0.76	<0.001
IFNα response	2.12	0.83	0.001

b

MSS samples of GSE39582

Gene sets	NES	ES	FDR
Allograft rejection	2.37	0.72	<0.001
IFNγ response	2.35	0.79	<0.001
Complement	2.29	0.64	<0.001
Inflammatory response	2.25	0.68	<0.001
KRAS signaling	2.25	0.61	<0.001
IFNα response	2.20	0.81	<0.001
IL-6/JAK/STAT3 response	2.14	0.67	<0.001
TNFα response	2.10	0.70	0.001

MSI samples of GSE39582

Gene sets	NES	ES	FDR
Allograft rejection	2.43	0.78	<0.001
Complement	2.29	0.64	<0.001
Inflammatory response	2.28	0.69	<0.001
IL-6/JAK/STAT3 response	2.26	0.72	<0.001
IFNγ response	2.24	0.84	<0.001
IFNα response	2.16	0.89	0.002

c

MSS samples of GSE41258

Gene sets	NES	ES	FDR
Allograft rejection	2.12	0.63	0.002
IFNγ response	2.04	0.74	<0.001

MSI samples of GSE41258

Gene sets	NES	ES	FDR
IFNγ response	2.15	0.76	0.003
IFNα response	2.03	0.79	0.011

d

MSS samples of GSE26682

Gene sets	NES	ES	FDR
IFNγ response	2.25	0.76	<0.001
Allograft rejection	2.22	0.68	<0.001
IFNα response	2.20	0.80	<0.001

MSI samples of GSE26682

Gene sets	NES	ES	FDR
Allograft rejection	2.20	0.69	0.002
IFNγ response	2.15	0.74	0.002
IFNα response	2.15	0.81	0.002

e

MSS samples of pooled_cohort_1

Gene sets	NES	ES	FDR
IFNγ response	2.28	0.77	<0.001
Allograft rejection	2.22	0.69	<0.001
IFNα response	2.10	0.77	0.001
Inflammatory response	2.08	0.63	0.001
IL-6/JAK/STAT3 response	2.02	0.63	0.003

MSI samples of pooled_cohort_1

Gene sets	NES	ES	FDR
Allograft rejection	2.38	0.77	<0.001
IFNγ response	2.29	0.82	<0.001
IFNα response	2.24	0.89	<0.001
Inflammatory response	2.15	0.64	0.001
Complement	2.15	0.63	0.001
IL-6/JAK/STAT3 response	2.08	0.66	0.002

f

MSS samples of pooled_cohort_2

Gene sets	NES	ES	FDR
Allograft rejection	2.11	0.68	0.003
IFNγ response	2.07	0.76	0.001
IL-6/JAK/STAT3 response	2.07	0.67	0.001
Complement	2.07	0.60	0.002
Inflammatory response	2.06	0.64	0.001
IFNα response	2.00	0.77	0.001

MSI samples of pooled_cohort_2

Gene sets	NES	ES	FDR
Allograft rejection	2.09	0.73	0.002
Complement	2.05	0.62	0.004
IFNγ response	1.98	0.80	0.005

Figure S5: **(a-f)** Gene set enrichment analysis (GSEA) showed the significant functional gene sets enriched in the GSE39582, COAD, GSE41258, GSE26682, pooled _cohort_1 and pooled_cohort_2, respectively.

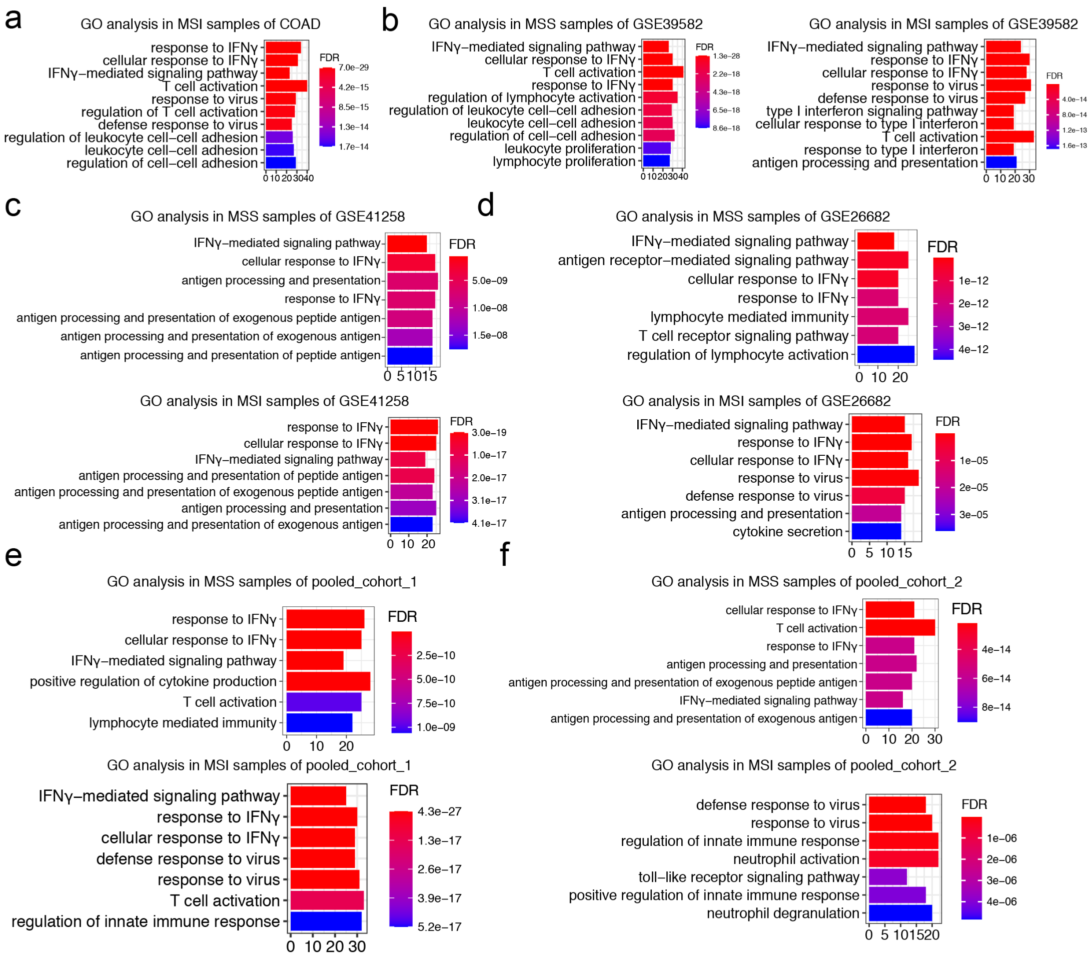

Figure S6: **(a-f)** GO analysis based on the top 200 differential expressed genes that were screened between high versus low GBP2 expression separated by median expression of GBP2 in the GSE39582, COAD, GSE41258, GSE26682, pooled _cohort_1 and pooled_cohort_2, respectively.

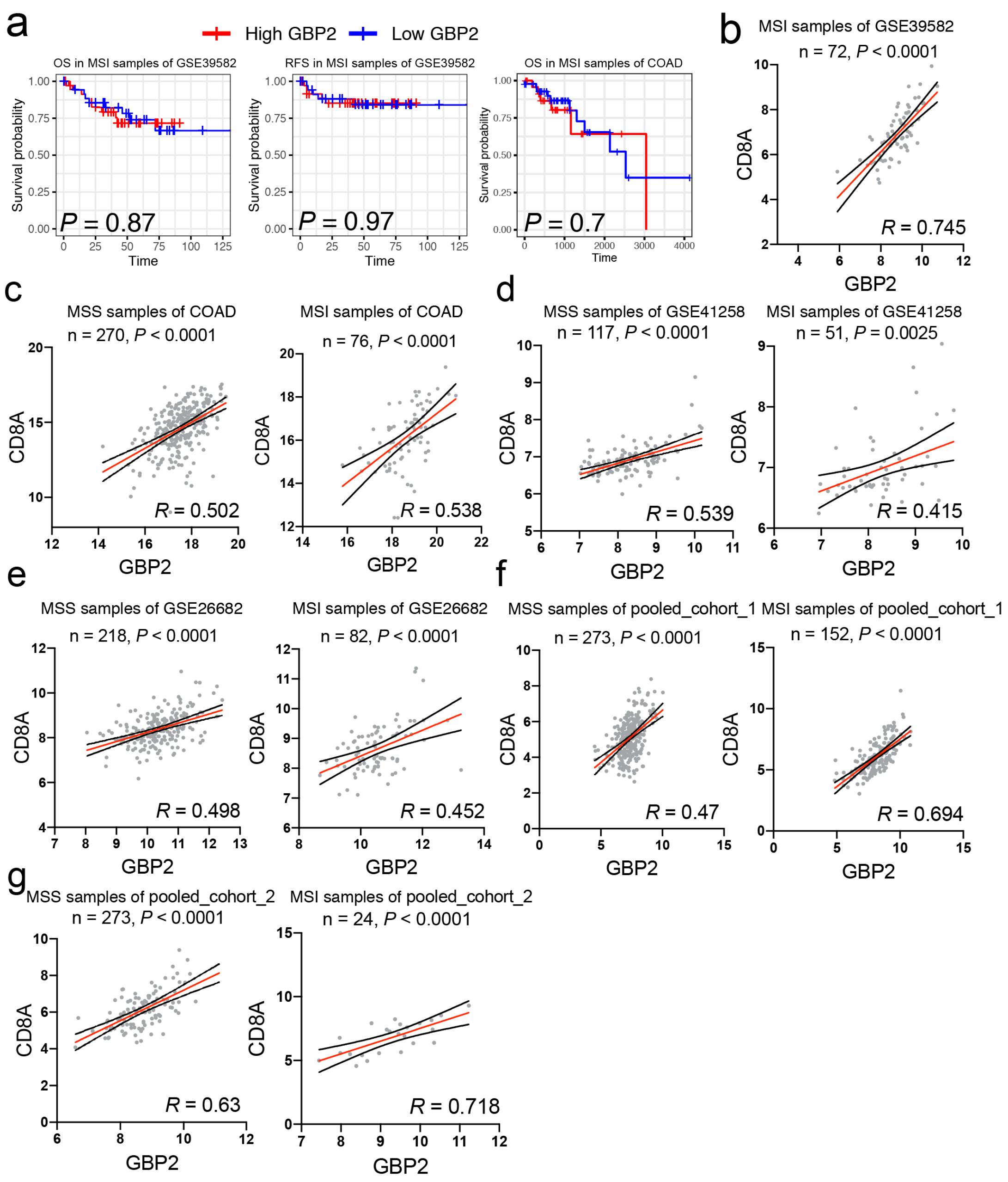

Figure S7: **(a)** Survival analysis showing the relationship between GBP2 levels and the OS and RFS of MSI samples in GSE39582 and COAD. (**b-g**) The correlation between GBP2 and CD8A in the GSE39582, COAD, GSE41258, GSE26682, pooled _cohort_1 and pooled_cohort_2, respectively.

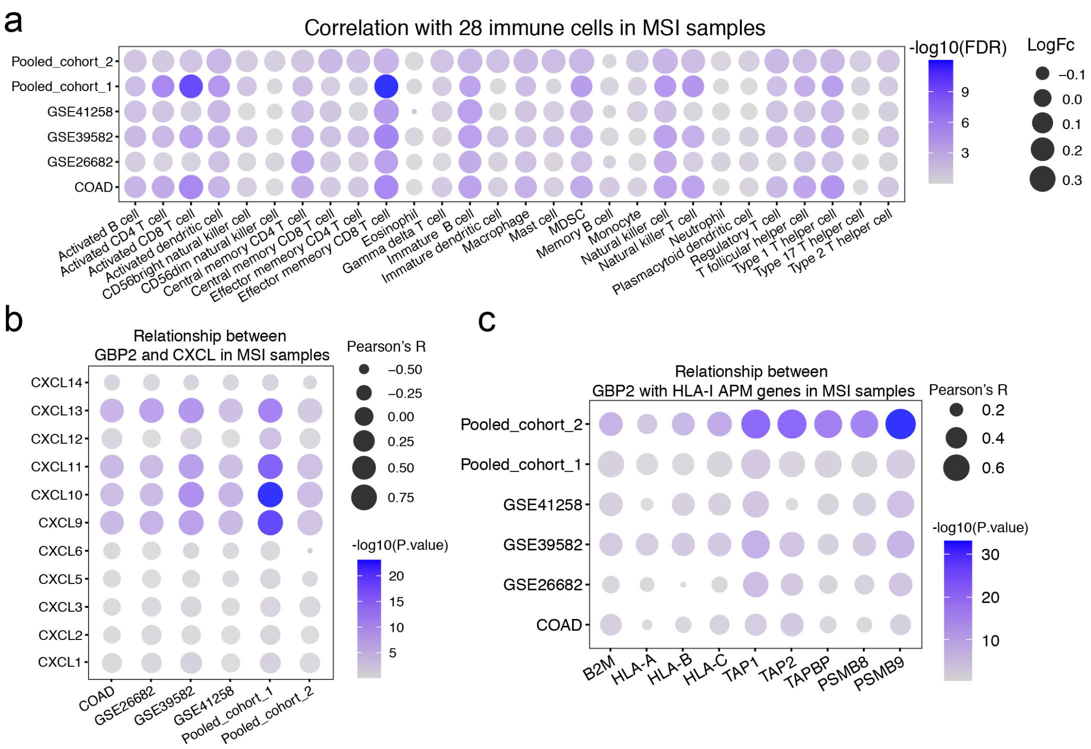

Figure S8: **(a)** Bubble plot representation showing the correlation between GBP2 expression and 28 immune cells in MSI samples of the six cohort. **(b)** Bubble plot representation showing the correlation between GBP2 and CXCL genes in MSI samples of the six cohort. **(c)** Bubble plot representation showing the correlation between GBP2 and HLA-I APM genes in MSI samples of the six cohort.

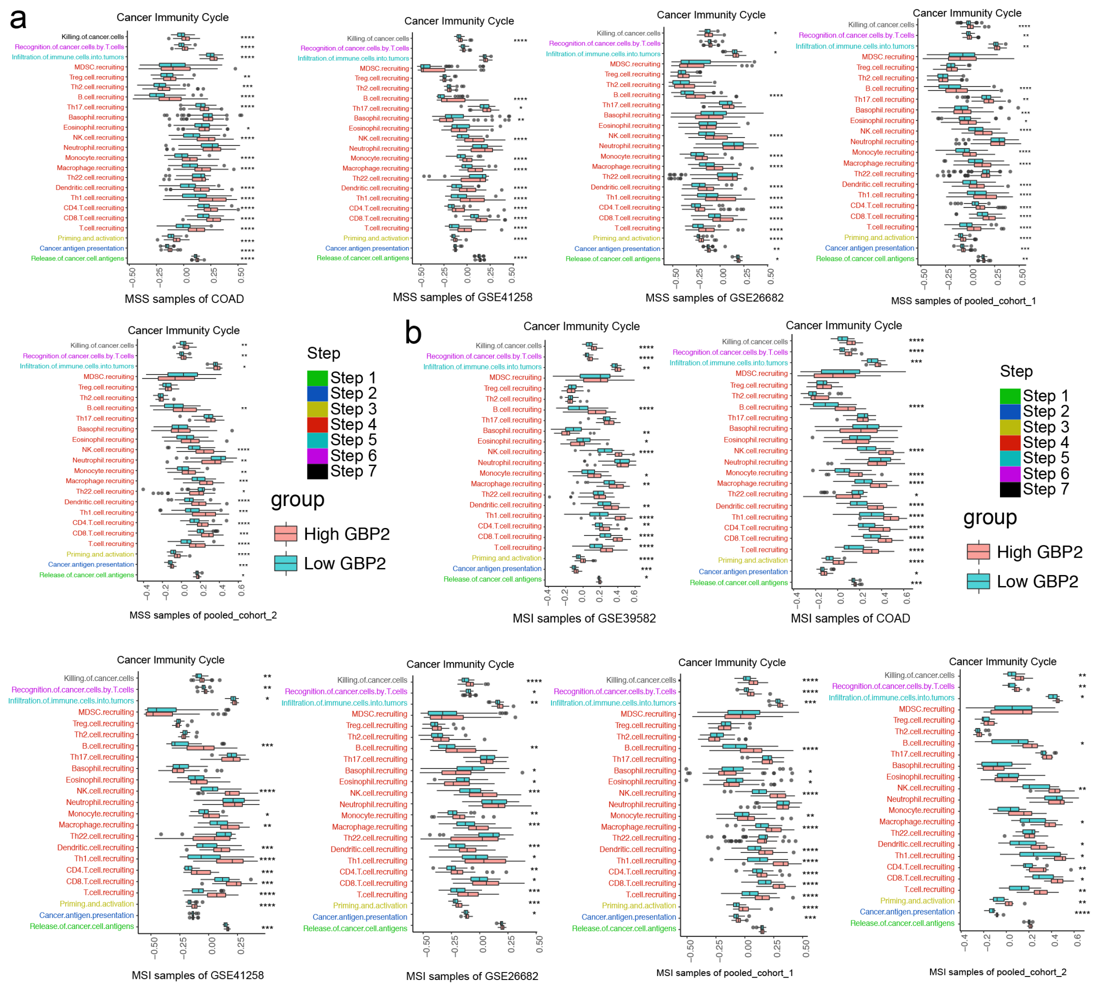

Figure S9: **(a-b)** Differences in the various steps of the cancer immunity cycle between high- and low-GBP2 groups in the MSS and MSI samples of GSE39582, COAD, GSE41258, GSE26682, pooled _cohort_1 and pooled_cohort_2, respectively. *$P < 0.05$, **$P < 0.01$, ***$P < 0.001$, ****$P < 0.0001$ versus control group.

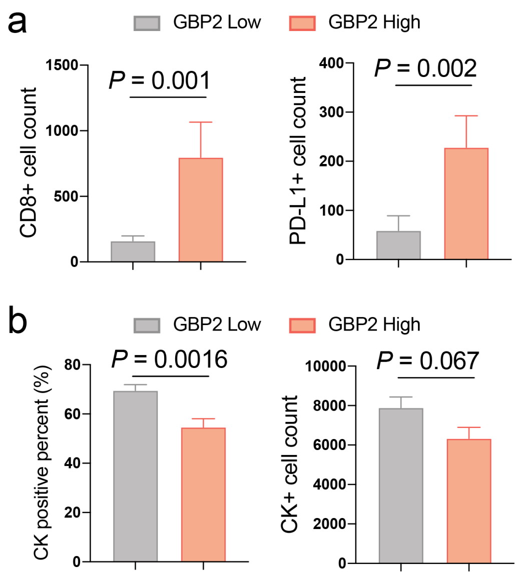

Figure S10: (a) Box plots showing the cell counts of CD8 and PD-L1 between the high and low GBP2 expression group of the TMA cohort. **(b)** Box plots showing the positive percent and cell count of CK between the high and low GBP2 expression group of the TMA cohort.

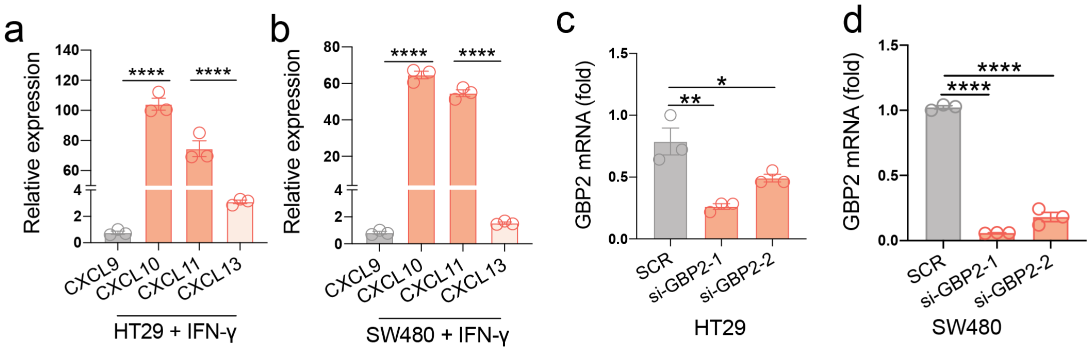

Figure S11: The difference expression levels of CXCL9, 10, 11 and 13 under the IFN-γ treatment in HT29 **(a)** and SW480 cells **(b)** by PCR, respectively. Real-time PCR used to analyze the mRNA expression levels of GBP2 in HT29 cells **(c)** and SW480 cells **(d)** transfected with SCR and siRNA (si-1, si-2). **P* < 0.05, ***P* < 0.01, *****P* < 0.0001 versus control group.

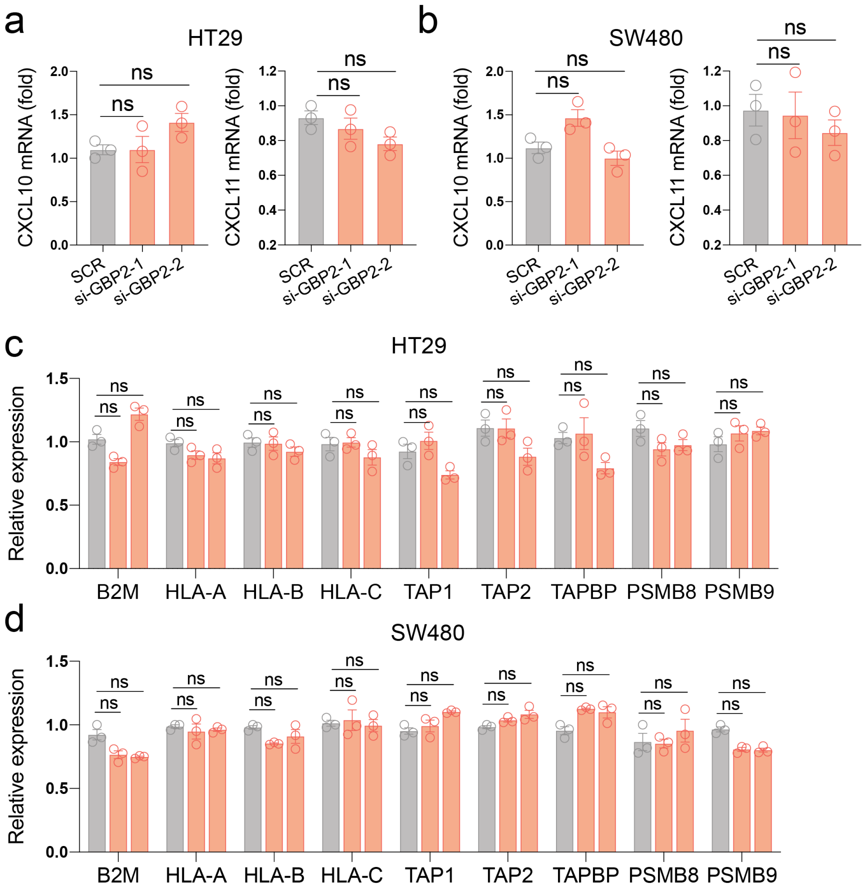

Figure S12: Real-time PCR were used to analyze the mRNA expression level of CXCL10 and CXCL11 in HT29 cells **(a)** and SW480 cells **(b)** transfected with SCR and GBP2 siRNA (si-1, si-2). Real-time PCR were used to analyze the mRNA expression level antigen processing machinery in HT29 cells **(c)** and SW480 cells **(d)** transfected with SCR and GBP2 siRNA (si-1, si-2). ns, not significant; SCR, scramble.

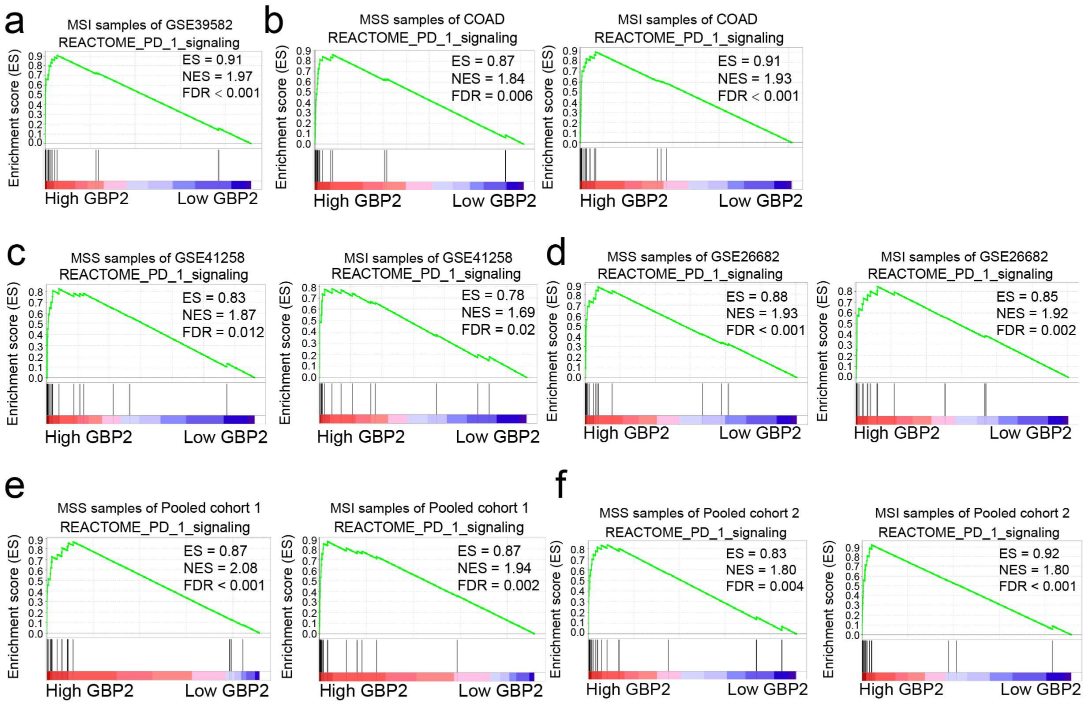

Figure S13: (a-e) GSEA plots of reactome PD-1 signaling and showing positively correlation with lower expression of GBP2 in the COAD, GSE41258, GSE26682, pooled _cohort_1 and pooled_cohort_2, respectively.

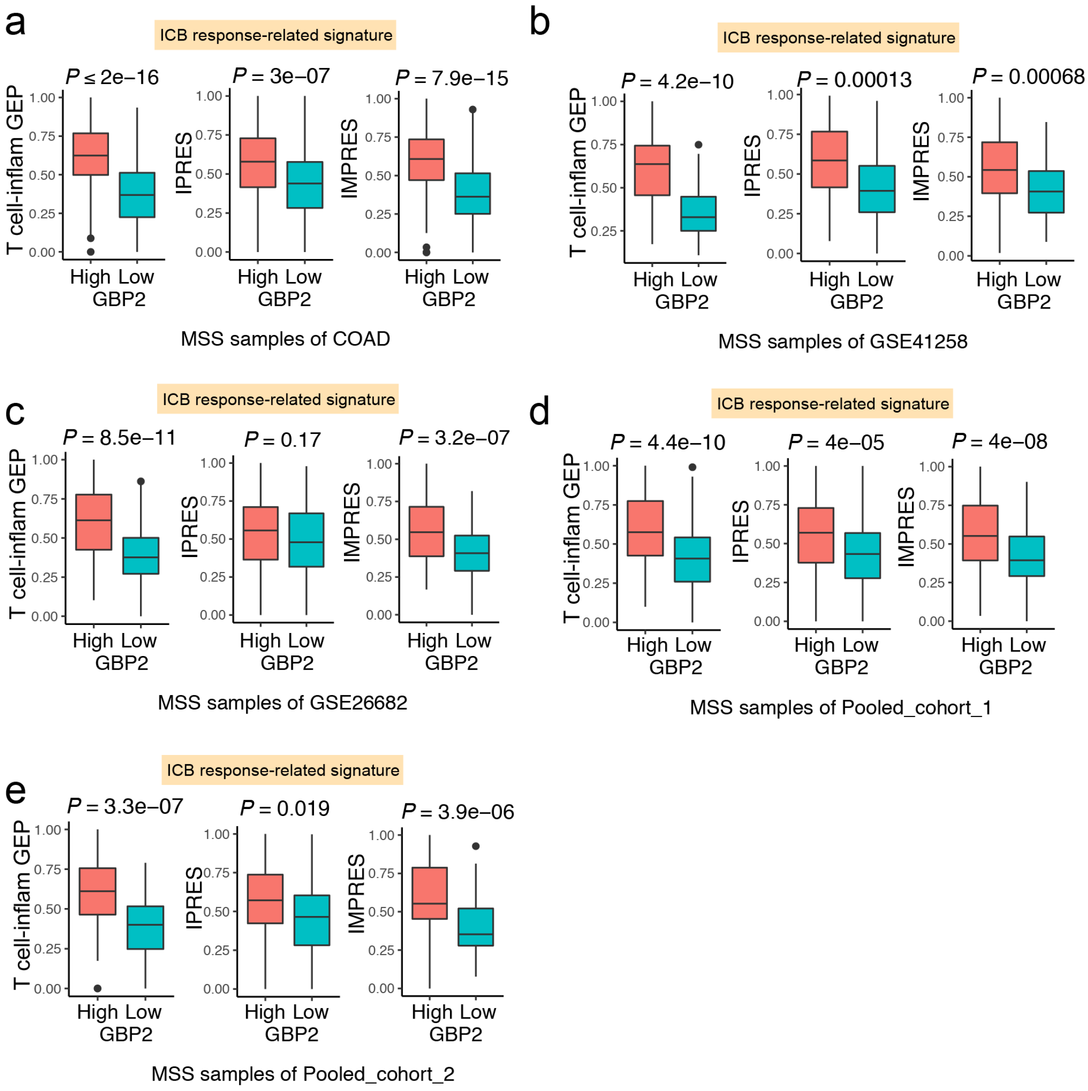

Figure S14: (a-e) Box plots showing expression of ICB response-related signatures between high and low GBP2 group in the COAD, GSE41258, GSE26682, pooled _cohort_1 and pooled_cohort_2, respectively.

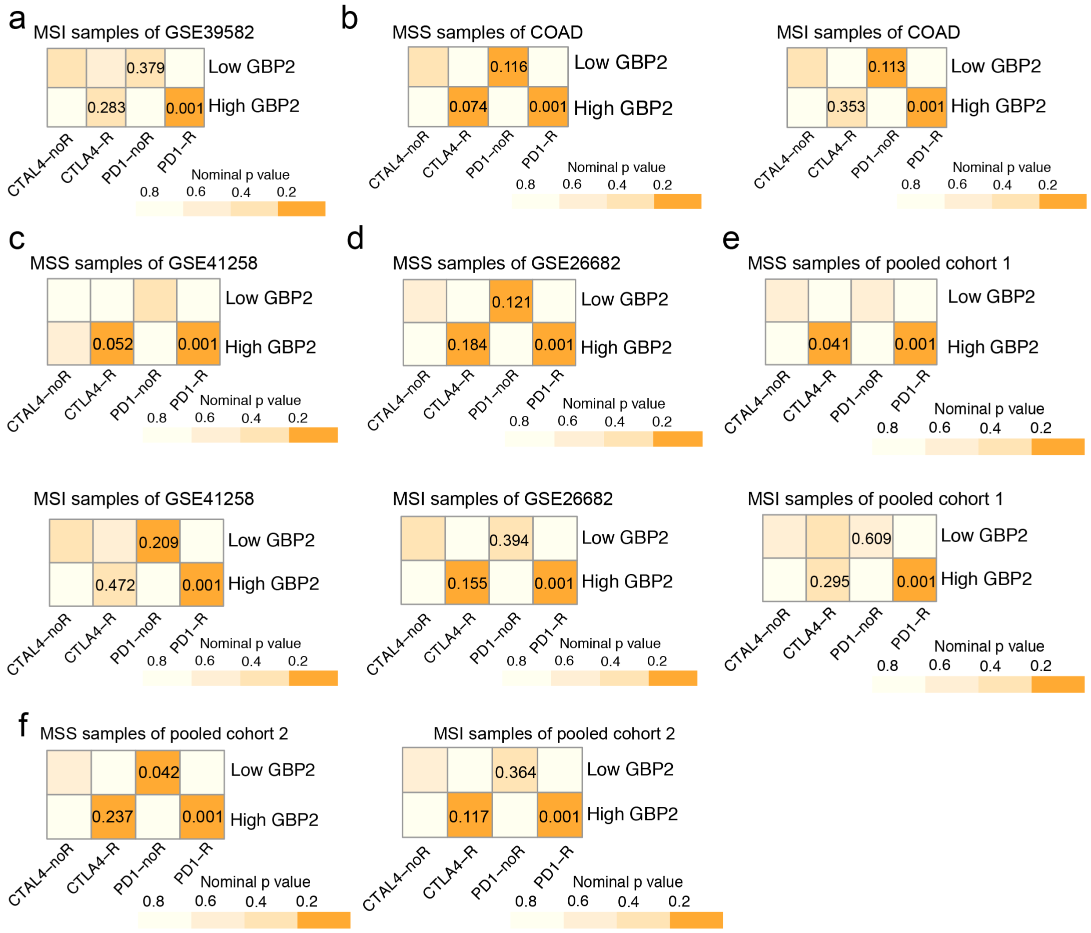

Figure S15: (a-e) Submap analysis demonstrates that the high GBP2 group in the other five CRC cohort are nearly identical to the PD1-response (PD1-R) group defined in the melanoma cohort

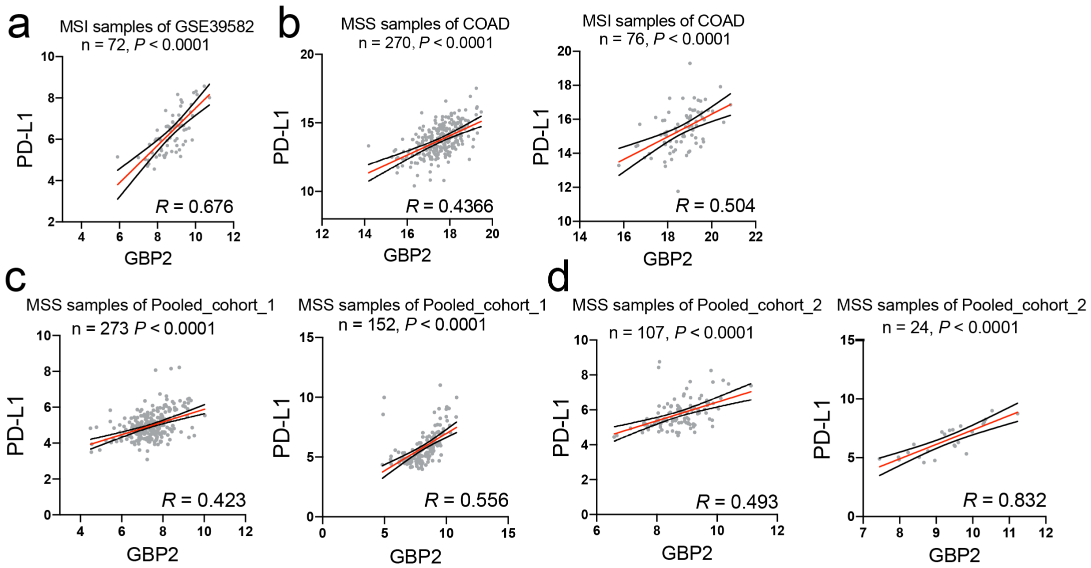

Figure S16: (a-d) The correlation between GBP2 and PD-L1 in the GSE39582, COAD, pooled_cohort_1 and pooled_cohort_2 cohorts, respectively.

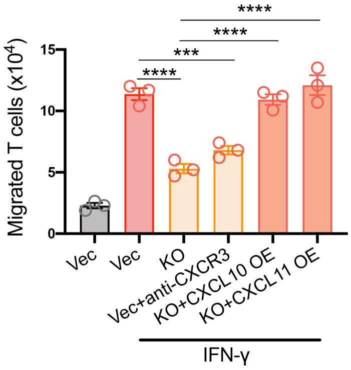

Figure S17: Deletion of GBP2 impaired T cell attraction by Transwell assay. Vec or GBP2-KO CT26 cells were treated with IFN-γ for 24 hours, and conditioned medium (CM) was collected.
