# Supplementary material for: Subtyping of microsatellite stability colorectal cancer reveals guanylate binding protein 2 (GBP2) as a potential immunotherapeutic target
Source: J Immunother Cancer. 2022 Apr 5;10(4):e004302. doi: 10.1136/jitc-2021-004302 (PMC8984016; doi:10.1136/jitc-2021-004302)
Supplement: Supplementary data [file jitc-2021-004302supp002.pdf]

Table S1. Clinical cohorts used in this study.

Cohort	Sample description	platform
COAD	270 MSS CRC samples	Illumina HiSeq 2000
	76 MSI CRC samples	
GSE26682	218 MSS CRC samples	GPL96+GPL570
	82 MSI CRC samples	
GSE41258	117 MSS CRC samples	GPL96
	51 MSI CRC samples	
GSE39582	439 pMMR CRC samples	GPL570
	72 dMMR CRC samples	
Tissue microarray	62 MSS CRC samples	--
Pooled cohort 1	273 MSS CRC samples	GPL570
(GSE4554+GSE13067+	152 MSI CRC samples	
GSE13294+GSE18088+		
GSE75316)		
Pooled cohort 2	107 MSS CRC samples	GPL570
(GSE39084+GSE35896)	24 MSI CRC samples	
