# Supplementary material for: Subtyping of microsatellite stability colorectal cancer reveals guanylate binding protein 2 (GBP2) as a potential immunotherapeutic target
Source: J Immunother Cancer. 2022 Apr 5;10(4):e004302. doi: 10.1136/jitc-2021-004302 (PMC8984016; doi:10.1136/jitc-2021-004302)
Supplement: Supplementary data [file jitc-2021-004302supp004.pdf]

Table S3. Publicly available gene signatures selected in this study.

Signature Name	References
Immune enrichment score	Yoshihara et al. Nat Commun. 2013; 4: 2612
Stromal enrichment score	Yoshihara et al. Nat Commun. 2013; 4: 2612
Immune cell subsets	Cancer Genome Atlas Network. Cell. 2015; 161: 1681-96
Immune signalling molecules	Cancer Genome Atlas Network. Cell. 2015; 161: 1681-96
13 T-cell signature	Spranger et al. Proc Natl Acad Sci U S A. 2016;113(48): E7759-E7768.
T cells	Iglesia et al. Clin Cancer Res. 2014;20(14):3818–3829.
CD8 T cells	Iglesia et al. Clin Cancer Res. 2014;20(14):3818–3829.
Cytotoxic cells	Bindea et al. Immunity. 2013; 39: 782-95
6-gene IFN-γ signature	Chow et al. J Clin Oncol. 34, (suppl; abstr 6010) 2016
CYT	Rooney et al. Cell. 2015; 160: 48-61
Chemokines	Coppola et al. Am J Pathol. 2011; 179(1): 37–45.
HLA	He et al. J Exp Clin Cancer Res. 2018;37(1):327.
Checkpoints	He et al. J Exp Clin Cancer Res. 2018;37(1):327.
TIL	He et al. J Exp Clin Cancer Res. 2018;37(1):327.
IPRES	Hugo W et al. Cell. 2016 Mar 24;165(1):35-44
IMPRES	Auslander N et al. Nat Med. 2018 Oct;24(10):1545-1549.
T cell-inflamed GEP	Cristescu et al. Science. 2018; 362(6411): eaar3593.
