# Supplementary material for: Subtyping of microsatellite stability colorectal cancer reveals guanylate binding protein 2 (GBP2) as a potential immunotherapeutic target
Source: J Immunother Cancer. 2022 Apr 5;10(4):e004302. doi: 10.1136/jitc-2021-004302 (PMC8984016; doi:10.1136/jitc-2021-004302)
Supplement: Supplementary data [file jitc-2021-004302supp006.pdf]

Table S8. The characteristics of patients in the COAD dataset according to the high and low *GBP2* expression group.

Variables	*GBP2* positive percent			*P* value
	Total (n = 346)	High (n = 173)	Low (n = 173)	
Gender				
Male, (%)	190 (54.9)	93 (53.8)	97 (56.1)	0.666
Female, (%)	156 (45.1)	80 (46.2)	76 (43.9)	
Age				
< 65 years, (%)	131 (37.9)	63 (36.4)	68 (39.3)	0.579
≥ 65 years, (%)	215 (62.1)	110 (63.6)	105 (60.7)	
History_of_colon_polyps				
No, (%)	188 (54.3)	90 (52.0)	98 (56.6)	0.572
Yes, (%)	107 (30.9)	58 (33.5)	49 (28.3)	
NA, (%)	51 (14.7)	25 (14.5)	26 (15.0)	
Lymphatic_invasion				
No, (%)	192 (55.5)	102 (59.0)	90 (52.0)	0.164
Yes, (%)	125 (36.1)	61 (35.2)	64 (37.0)	
NA, (%)	29 (8.4)	10 (5.8)	19 (11.0)	
Venous_invasion				
No, (%)	232 (67.1)	114 (65.9)	118 (68.2)	0.897
Yes, (%)	70 (20.2)	36 (20.8)	34 (19.7)	
NA, (%)	44 (12.7)	23 (13.3)	21 (12.1)	
T stage				
Tis+T1+T2, (%)	70 (20.2)	39 (22.5)	31 (17.9)	0.314
T3, (%)	241 (69.7)	120 (69.4)	121 (69.9)	
T4, (%)	35 (10.1)	14 (8.1)	21 (12.1)	
N stage				
N0, (%)	203 (58.7)	110 (63.6)	93 (53.8)	**0.036**
N1, (%)	81 (23.4)	41 (23.7)	40 (23.1)	
N2, (%)	62 (17.9)	22 (12.7)	40 (23.1)	
M stage				
M0, (%)	264 (76.3)	140 (80.9)	124 (71.7)	**0.017**
M1, (%)	43 (12.4)	12 (6.9)	31 (17.9)	
Mx, (%)	33 (9.5)	18 (10.4)	15 (8.7)	
NA, (%)	6 (1.7)	3 (1.7)	3 (1.7)	
TNM stage				
I, (%)	59 (17.1)	36 (20.8)	23 (13.3)	**0.010**
II, (%)	131 (37.9)	72 (41.6)	59 (34.1)	
III, (%)	103 (29.8)	49 (28.3)	54 (31.2)	
IV, (%)	43 (12.4)	12 (6.9)	31 (17.9)	
NA, (%)	10 (2.8)	4 (2.3)	6 (3.5)	

Abbreviations: NA, not available;
