# Supplementary material for: Subtyping of microsatellite stability colorectal cancer reveals guanylate binding protein 2 (GBP2) as a potential immunotherapeutic target
Source: J Immunother Cancer. 2022 Apr 5;10(4):e004302. doi: 10.1136/jitc-2021-004302 (PMC8984016; doi:10.1136/jitc-2021-004302)
Supplement: Supplementary data [file jitc-2021-004302supp007.pdf]

**Table S9.** The characteristics of patients in the GSE41258 dataset according to the high and low *GBP2* expression group.

| Variables | *GBP2* positive percent | | | *P* value |
|---|---|---|---|---|
| | Total (n = 168) | High (n = 84) | Low (n = 84) | |
| Gender | | | | |
| Male, (%) | 83 (49.4) | 44 (52.4) | 39 (46.4) | 0.440 |
| Female, (%) | 85 (50.6) | 40 (47.6) | 45 (53.6) | |
| Age | | | | |
| < 65 years, (%) | 77 (45.8) | 41 (48.8) | 36 (42.9) | 0.439 |
| ≥ 65 years, (%) | 91 (54.2) | 43 (51.2) | 48 (57.1) | |
| T stage | | | | |
| T1+T2, (%) | 38 (22.6) | 16 (19.0) | 22 (26.2) | 0.269 |
| T3+T4, (%) | 130 (77.4) | 68 (81.0) | 62 (73.8) | |
| N stage | | | | |
| N0, (%) | 85 (50.6) | 43 (51.2) | 42 (50.0) | 0.982 |
| N1, (%) | 42 (25.0) | 21 (25.0) | 21 (25.0) | |
| N2, (%) | 41 (24.4) | 20 (23.8) | 21 (25.0) | |
| M stage | | | | |
| M0, (%) | 116 (69.0) | 64 (76.2) | 52 (61.9) | **0.045** |
| M1, (%) | 52 (31.0) | 20 (23.8) | 32 (38.1) | |
| TNM stage | | | | |
| I, (%) | 28 (16.7) | 13 (15.5) | 15 (17.9) | 0.161 |
| II, (%) | 41 (24.4) | 24 (28.6) | 17 (20.2) | |
| III, (%) | 47 (28.0) | 27 (32.1) | 20 (23.8) | |
| IV, (%) | 52 (31.0) | 20 (23.8) | 32 (38.1) | |
