# Supplementary material for: Subtyping of microsatellite stability colorectal cancer reveals guanylate binding protein 2 (GBP2) as a potential immunotherapeutic target
Source: J Immunother Cancer. 2022 Apr 5;10(4):e004302. doi: 10.1136/jitc-2021-004302 (PMC8984016; doi:10.1136/jitc-2021-004302)
Supplement: Supplementary data [file jitc-2021-004302supp008.pdf]

Table S10. The characteristics of MSS patients in the tissue microarray according *GBP2* positive percent.

Variables	*GBP2* positive percent			*P* value
	Total (n = 62)	High (n = 31)	Low (n = 31)	
Gender				
Male, (%)	33 (53.2)	13 (41.9)	20 (64.5)	0.128
Female, (%)	29 (46.8)	18 (58.1)	11 (35.5)	
Age				
< 65 years, (%)	23 (37.1)	12 (38.7)	11 (35.5)	1
≥ 65 years, (%)	39 (62.9)	19 (61.3)	20 (64.5)	
Grade				
< grade II, (%)	17 (27.4)	8 (25.8)	9 (29.0)	1
≥ grade II, (%)	45 (72.6)	23 (74.2)	22 (71.0)	
T stage				
T1+2, (%)	3 (4.8)	1 (3.2)	2 (6.5)	**0.0386**
T3, (%)	48 (77.4)	28 (90.3)	20 (64.5)	
T4, (%)	11 (17.7)	2 (6.5)	9 (29.0)	
N stage				
N0, (%)	38 (61.3)	22 (71.0)	16 (51.6)	0.0753
N1, (%)	18 (29.0)	5 (16.1)	13 (41.9)	
N2, (%)	6 (9.7)	4 (12.9)	2 (6.5)	
M stage				
M0, (%)	62 (100)	31 (100)	31 (100)	NA
M1, (%)	0 (0)	0 (0)	0 (0)	
TNM stage				
I, (%)	3 (4.8)	1 (3.2)	2 (6.5)	0.2298
II, (%)	35 (56.5)	21 (67.7)	14 (45.2)	
III, (%)	25 (38.7)	9 (29.0)	15 (48.4)	

Abbreviations: NA, not available;
