# Supplementary material for: Subtyping of microsatellite stability colorectal cancer reveals guanylate binding protein 2 (GBP2) as a potential immunotherapeutic target
Source: J Immunother Cancer. 2022 Apr 5;10(4):e004302. doi: 10.1136/jitc-2021-004302 (PMC8984016; doi:10.1136/jitc-2021-004302)
Supplement: Supplementary data [file jitc-2021-004302supp009.pdf]

Table S11. The characteristics of patients in the GSE39582 dataset according to the high and low *GBP2* expression group.

Variables	*GBP2* positive percent			*P* value
	Total (n = 511)	High (n = 256)	Low (n = 255)	
Gender				
Male, (%)	286 (56.0)	142 (55.5)	144 (56.5)	0.820
Female, (%)	225 (44.0)	114 (44.5)	111 (43.5)	
Age				
< 65 years, (%)	194 (38.0)	92 (35.9)	102 (40.0)	0.387
≥ 65 years, (%)	316 (61.8)	163 (63.7)	153 (60.0)	
NA	1 (0.2)	1 (0.4)	0 (0)	
T stage				
T0+T1+T2, (%)	55 (10.8)	28 (10.9)	27 (10.6)	1
T3+T4, (%)	433 (84.7)	216 (84.4)	217 (85.1)	
Tis, (%)	3 (0.6)	2 (0.8)	1 (0.4)	
NA, (%)	20 (3.9)	10 (3.9)	10 (3.9)	
N stage				
N0+N1, (%)	383 (75.0)	188 (73.4)	195 (76.5)	0.883
N2+N3, (%)	102 (20.0)	55 (21.5)	47 (18.4)	
N+, (%)	6 (1.2)	3 (1.2)	3 (1.2)	
NA, (%)	20 (3.9)	10 (3.9)	10 (3.9)	
M stage				
M0, (%)	431 (84.3)	217 (84.8)	214 (83.9)	0.641
M1, (%)	58 (11.4)	27 (10.5)	31 (12.2)	
Mx, (%)	2 (0.4)	2 (0.8)	0 (0)	
NA, (%)	20 (3.9)	10 (3.9)	10 (3.9)	
TNM stage				
0+I+II, (%)	255 (49.9)	132 (51.6)	123 (48.2)	0.452
III+IV, (%)	256 (50.1)	124 (48.4)	132 (51.8)	
Chemotherapy				
No, (%)	278 (54.4)	147 (57.4)	131 (51.4)	0.522
Yes, (%)	217 (42.5)	105 (39.8)	112 (45.1)	
NA, (%)	16 (3.1)	7 (2.7)	9 (3.5)	

Abbreviations: NA, not available;
