# Supplementary material for: Subtyping of microsatellite stability colorectal cancer reveals guanylate binding protein 2 (GBP2) as a potential immunotherapeutic target
Source: J Immunother Cancer. 2022 Apr 5;10(4):e004302. doi: 10.1136/jitc-2021-004302 (PMC8984016; doi:10.1136/jitc-2021-004302)
Supplement: Supplementary data [file jitc-2021-004302supp012.pdf]

Table S6. Sequences of primers for PCR.

Gene		Real-time PCR
Homo-GAPDH	5'Primer	GTCTCCTCTGACTTCAACAGCG
	3'Primer	ACCACCCTGTTGCTGTAGCCAA
Homo-GBP2	5'Primer	GTTCCTACATCCTCAGCCATTCC
	3'Primer	CCACTGCTGATGGCATTGACGT
Homo-CXCL9	5'Primer	CTGTTCCTGCATCAGCACCAAC
	3'Primer	TGAACTCCATTCTTCAGTGTAGCA
Homo-CXCL10	5'Primer	GGTGAGAAGAGATGTCTGAATCC
	3'Primer	GTCCATCCTTGGAAGCACTGCA
Homo-CXCL11	5'Primer	AAGGACAACGATGCCTAAATCCC
	3'Primer	CAGATGCCCTTTTCCAGGACTTC
Homo-CXCL13	5'Primer	TATCCCTAGACGCTTCATTGATCG
	3'Primer	CCATTCAGCTTGAGGGTCCACA
Homo-B2M	5'Primer	CCACTGAAAAAGATGAGTATGCCT
	3'Primer	CCAATCCAAATGCGGCATCTTCA
Homo-HLA-A	5'Primer	AGATACACCTGCCATGTGCAGC
	3'Primer	GATCACAGCTCCAAGGAGAACC
Homo-HLA-B	5'Primer	CTGCTGTGATGTGTAGGAGGAAG

	3’Primer	GCTGTGAGAGACACATCAGAGC
Homo-HLA-C	5’Primer	GGAGACACAGAAGTACAAGCGC
	3’Primer	ACATCCTCTGGAGGGTGTGAGA
Homo-TAP1	5’Primer	GCAGTCAACTCCTGGACCACTA
	3’Primer	CAAGGTTCCCACTGCTTACAGC
Homo-TAP2	5’Primer	ATGCCCTTCACAATAGCAGCGG
	3’Primer	CCAAAACTGCGAACGGTCTGCA
Homo-TAPBP	5’Primer	GAGCCTGTTCTCATCACCATGG
	3’Primer	GTAGGCAAAGCTCAAGTCCAGC
Homo-PSMB8	5’Primer	CCTTACCTGCTTGGCACCATGT
	3’Primer	TTGGAGGCTGCCGACACTGAAA
Homo-PSMB9	5’Primer	CGAGAGGACTTGTCTGCACATC
	3’Primer	CACCAATGGCAAAAGGCTGTCG
